# Dynamic helical cyclophanes with two quadruply-bridged planes arranged in an “obverse and/or reverse” relation†
†Electronic supplementary information (ESI) available: X-ray and energy-minimized structures, NMR and CD spectroscopic data, and experimental details of new compound preparation. CCDC 1439417. For ESI and crystallographic data in CIF or other electronic format see DOI: 10.1039/c5sc04673d


**DOI:** 10.1039/c5sc04673d

**Published:** 2016-01-29

**Authors:** Ryo Katoono, Shunsuke Kawai, Takanori Suzuki

**Affiliations:** a Department of Chemistry , Faculty of Science , Hokkaido University , Sapporo 060-0810 , Japan . Email: katoono@sci.hokudai.ac.jp ; Fax: +81 11 706 2714 ; Tel: +81 11 706 3396

## Abstract

We describe the design of two types of cyclophanes that generate dynamic helicity through the twisting of two planes in a clockwise or counterclockwise direction to give (*M*)- or (*P*)-helicity. We used a rectangular and anisotropic plane of 1,2,4,5-tetrakis(phenylethynyl)benzene (TPEB), since it can be stacked in pairs in two ways, in parallel or orthogonally, to be identified as distinct cyclophane molecules. We adopted a synthetic strategy for obtaining these two cyclophanes as a mixture using a macrocyclic intermediate that possessed two rotatable phenyl rings. We introduced necessary parts into the rotators to give a mixture of rotational isomers leading to a parallel or orthogonal arrangement of TPEBs, and then doubly bridged two planes of TPEB to form quadruply-bridged cyclophanes. We consider that such two planes in each cyclophane are in an “obverse and/or reverse” relation. In each cyclophane, we found unique dynamic helical forms with (*M*)- or (*P*)-helicity as well as an inherently non-chiral form. Normally, the screw-sense preference of dynamic helicity would be controlled through the intramolecular or supramolecular transmission of central chirality, when a chiral auxiliary is attached to the cyclophanes or a chiral guest is allowed to form a complex with the cyclophanes. In a case where two different substitution groups were used on bridging units to generate planar chirality in each cyclophane, the screw-sense preference was controlled through the arrangement of these substitution groups, and did not depend on the transmission of central chirality. Two different substitution groups desymmetrize the enantiomeric forms with (*M*)- or (*P*)-helicity generated in each dynamic helical cyclophane so that two dynamic helical forms with (*M*)- or (*P*)-helicity can be in a diastereomeric relation. Thus, a particular screw sense of dynamic helicity can be preferred, regardless of whether or not the two substitution groups possess some chiral element.

## Abstract 2

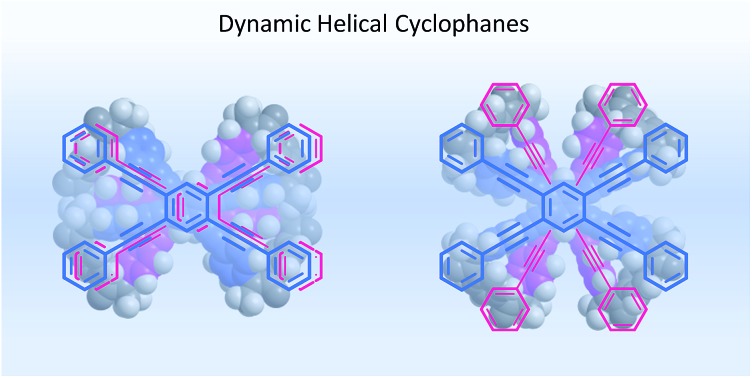
We describe the design of two types of cyclophanes that generate dynamic helicity through the twisting of two planes in a clockwise or counterclockwise direction to give (*M*)- or (*P*)-helicity.

## Introduction

**Scheme 1:**
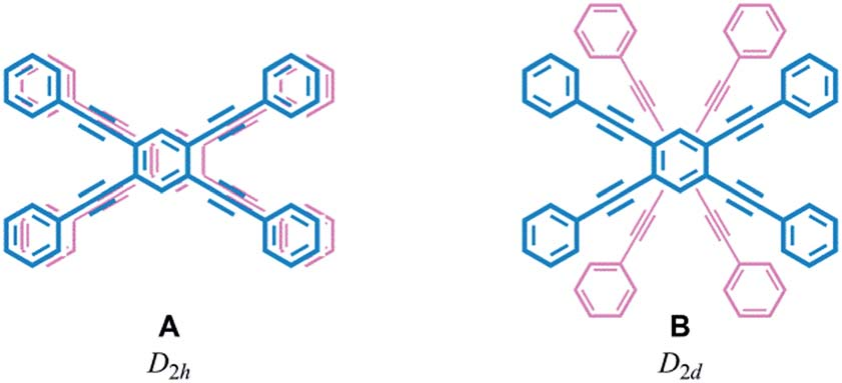
Two types of stacking of anisotropic planes (1,2,4,5-tetrasubstituted benzene).

**Fig. 1:**
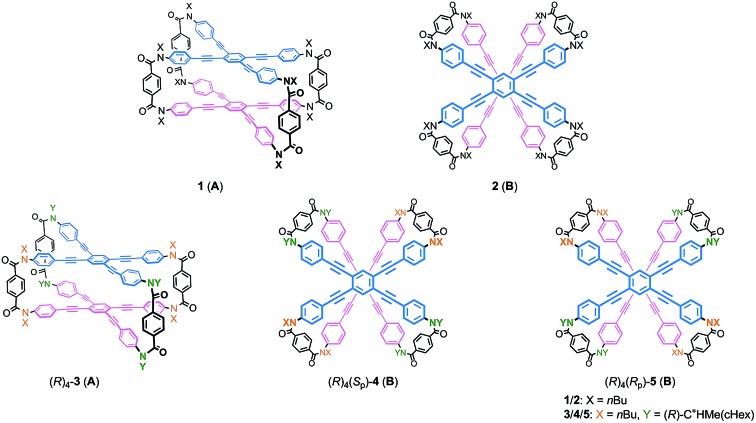
Chemical structures of dynamic helical cyclophanes **1-5** with two quadruply-bridged planes.

**Scheme 2:**
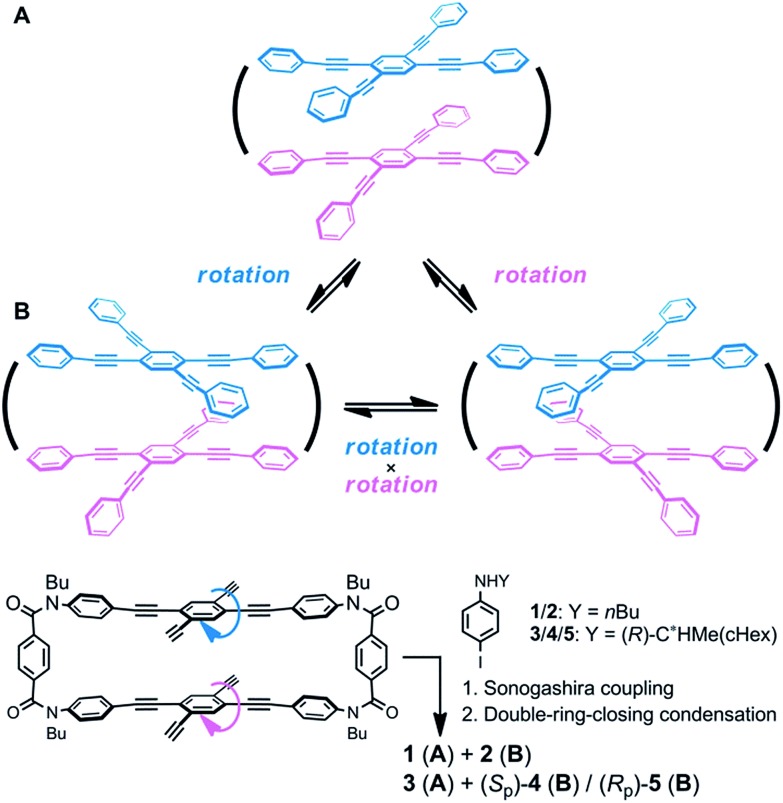
The “obverse and/or reverse” relation in two cyclophanes **A** and **B**, and a synthetic strategy for obtaining both quadruply-bridged cyclophanes **A** and **B**.

Helical twisting of achiral components that are stacked in a columnar assembly is a well-known method for creating helical architectures.^1^–^7^ The preference for a particular screw sense in the assembly is induced by the transmission of central chirality that exists in the periphery of an achiral component, or in external chiral additives. This methodology for generating and controlling helical chirality is suitable for layered structures in a molecule.^8^ In the case of supramolecular assemblies or in molecules with a layered structure, linear,^2^,8a,b,9a trigonal,^3^,8c,d,9b–e tetragonal,^4^,8e–g,9f hexagonal^5^ or more highly symmetric^6^ molecule(s) have often been used as an achiral component, and such regular polygonal symmetry would appear to provide a single manner of helical stacking. We were interested in a rectangular and anisotropic shape of 1,2,4,5-tetrasubstituted benzene, since it can be stacked in two distinct manners (Scheme 1).^10^ However, these two states might merge to be identical during twisting if the two planes are not fixed in relation to each other. Bridging of these two planes with multiple covalent bonds would enable the two states to be individualized as distinct cyclophane molecules.10a,^11^ We designed two types of cyclophanes **A** and **B** with two planes of 1,2,4,5-tetrakis(phenylethynyl)benzene (TPEB) that are stacked in pairs (Fig. 1). We used terephthalamide as a four-fold bridge in both types of cyclophanes. We synthesized the two types of cyclophanes **A** and **B** as a single mixture, and separated them by HPLC. As mentioned above, two planes are arranged in parallel (**A**) or orthogonally (**B**) (Scheme 1). To realize this relation in a cyclophane scaffold, we assumed an imaginary cyclophane with a two-fold bridge, in which one of the two diametrical axes is bridged with another on the upper and lower planes (Scheme 2). Rotation of a particular plane about the doubly bridged diametrical axis leads to isomerization between **A** and **B**, independent of whether such rotation is actually allowed or not. Double-rotation of the two planes leads to isomerization between the two enantiomeric forms of type **B**. If we consider this “obverse and/or reverse” relation between two planes, we can use a macrocyclic intermediate that possesses two rotatable phenyl rings. We introduced necessary parts into the rotators to give a mixture of rotational isomers, and then doubly bridged two planes to form quadruply-bridged cyclophanes **A** and **B** as a mixture (Scheme 2). The product ratio of these two cyclophanes should not significantly depend on the final ring-closing reaction even though their potential energies are different, but rather should depend on an earlier stage where at least one rotator is expected to be rotatable until the last part has been introduced.

**Scheme 3:**
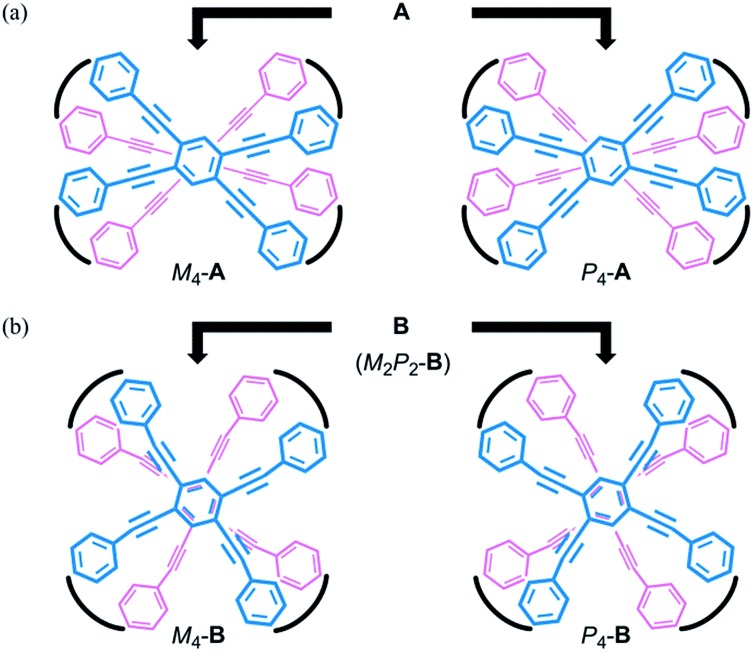
Generation of dynamic helicity through helical twisting of two planes in cyclophanes (a) **A** and (b) **B** with a four-fold bridge.

We envisioned that the helical twisting of two planes in each covalently bridged cyclophane could create unique dynamic helicity (Scheme 3). We designate the conformations of these cyclophanes as *M*_4_-**A**, *M*_2_*P*_2_-**B** and so on, where *M* and *P* denote the partial helicity that is generated between two bridged phenylethynyl groups. In cyclophane **A**, two planes would twist to create in concert four-fold partial helicity aligned in the same direction toward *M* (*M*_4_-**A**) or *P* (*P*_4_-**A**) (Scheme 3a). In cyclophane **B**, two planes are arranged orthogonally and thus are inherently twisted so that they can be considered a *meso*-like form (*M*_2_*P*_2_-**B**). Additional twisting is allowed by the inversion of partial helicity only at a particular two-fold bridge across the central benzene rings of TPEBs, and would lead to dynamic helical forms *M*_4_-**B** or *P*_4_-**B** (Scheme 3b). Note that *M*_4_-**A** and *M*_4_-**B** are different molecules, although they have been assigned the same symbol. Normally, the screw-sense preference of such dynamic helicity would be controlled through a transmission of central chirality,8b,c,^12^ as mentioned above.

**Scheme 4:**
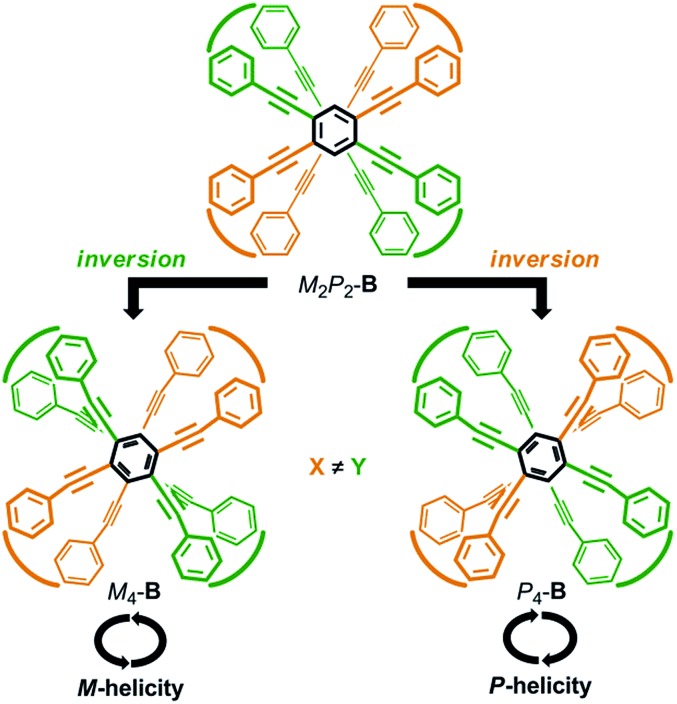
Generation of planar chirality and control of screw-sense preference of dynamic helicity through the arrangement of X and Y (X ≠ Y) on the bridging units (terephthalamide), which are drawn as an arc for clarity. Only a particular planar chiral enantiomer is depicted. If we assume that Y is the higher priority group, the stereochemistry of the depicted cyclophane (**B**) is *S*_p_.

Recently, we reported the design of a planar chiral cyclophane of type **A** through the differentiation of substitution groups X and Y (X ≠ Y) on each amide nitrogen of all four bridging units, and the control of screw-sense preference of dynamic helicity that was independent of any transmission of chirality.^13^ Here we demonstrate an alternative design with respect to type **B** (Scheme 4). We again used two different substitution groups X and Y (X ≠ Y). Two pairs of X are arranged at one bridge and another across the central benzene rings of TPEBs, and two pairs of Y are similarly arranged in the remaining two bridges. In cyclophanes of type **B**, the arrangement of X and Y generates planar chirality^14^ [(*S*_p_)-**4** (**B**) and (*R*_p_)-**5** (**B**)]. Such a planar chiral cyclophane is assured to be configurationally stable during dynamic interconversions among conformations. In an inherently twisted but *meso*-like form (*M*_2_*P*_2_-**B**), X is on a bridge with partial *M*-helicity and Y is on a bridge with partial *P*-helicity. The molecule is only allowed to deform once by inversion of the original partial helicity at a particular two-fold bridge of the four bridges. Deformation at two bridges with an X group would lead to the generation of a dynamic helical form with global (*P*)-helicity (*P*_4_-**B**). Another dynamic helical form (*M*_4_-**B**) with the contrary sense would be generated due to deformation at two bridges with a Y group. These two dynamic helical forms *M*_4_-**B** and *P*_4_-**B** are diastereomeric (X ≠ Y) and energetically nonequivalent. Thus, a particular screw sense of dynamic helicity would be preferred through the arrangement of X and Y. In a mirrored isomer with planar chirality, a contrary preference would be created by arrangement of the identical pair of X and Y (X ≠ Y).

## Results and discussion

### Synthesis of cyclophanes with two planes arranged in an “obverse and/or reverse” relation

**Fig. 2:**
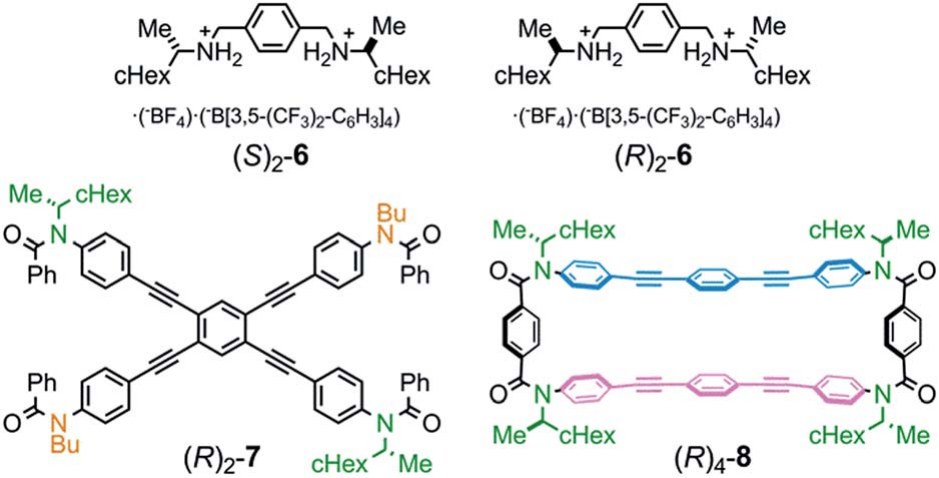
Chemical structures of chiral ditopic guests (*S*)_2_-**6**/(*R*)_2_-**6**, and references (*R*)_2_-**7** and (*R*)_4_-**8**.

We obtained the cyclophanes **1** (**A**) and **2** (**B**) in pure form (1/2 = 55 : 45) by HPLC separation (Schemes 2 and S1†). Due to the presence of mirrors in both **1** (*D*_2h_) and **2** (*D*_2d_), they are not chiral and were used to investigate the supramolecular transmission of central chirality upon complexation with a chiral guest. Alternatively, there is no mirror in the cyclophanes (*R*)_4_-**3**, (*R*)_4_-**4** and (*R*)_4_-**5**, due to the presence of central chirality (*R*) in the Y group. (*R*)_4_-**3** (**A**) is chiral but does not possess a chiral plane, and therefore it was used to investigate the intramolecular transmission of central chirality associated with the cyclophane. In cyclophanes of type **B**, planar chirality is inherently generated through the arrangement of X and Y (X ≠ Y). Only the differentiation and arrangement of X and Y are essential for producing planar chirality. Central chirality (*R*) in the Y group is not involved in the generation of planar chirality. A diastereomeric mixture of (*R*)_4_-**3**, (*R*)_4_-**4** and (*R*)_4_-**5** in a ratio of 62 : 25 : 13 was separated in this order by HPLC to give (*R*)_4_-**3** and (*R*)_4_-**4** in pure form, and (*R*)_4_-**5** as a mixture containing less than 6% (*R*)_4_-**4**. We did not determine the absolute configuration (*S*_p_) or (*R*_p_) with regard to the planar chirality of **4** and **5**, but arbitrarily assigned the second and third fractions to (*S*_p_)-**4** and (*R*_p_)-**5**, respectively, to describe the following results. As a chiral guest, we used diammonium salts8*c* (*S*)_2_-**6** and (*R*)_2_-**6** to investigate the supramolecular transmission of central chirality during complexation. As references, we prepared a single-layer TPEB derivative (*R*)_2_-**7**, and a doubly-bridged cyclophane (*R*)_4_-**8** (Fig. 2).

### Molecular structures of cyclophanes

**Fig. 3:**
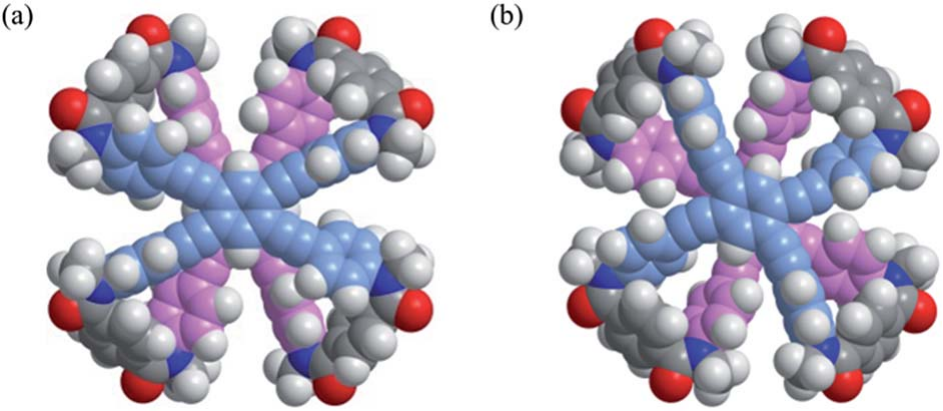
Energy-minimized structures for a model **2′** (**B**) [NMe], (a) *M*_2_*P*_2_-**2′** (rel. 0 kJ mol^–1^) and (b) *M*_4_-**2′** (+22.8 kJ mol^–1^), obtained by a conformational search using MacroModel software (v9.9 OPLS_2005, Monte Carlo Multiple Minimum method, non-solvated, 50 000 steps). Only a particular enantiomeric form with (*M*)-helicity is depicted.

A conformational search for a model **2′** (**B**) [X = Me] predicted that an inherently twisted but *meso*-like form (*M*_2_*P*_2_-**2′**) was the most energy-minimized structure (Fig. 3a), similar to that seen in a crystal.^15^ In addition, a global helical form *M*_4_-**2′** was also found at a higher energy level (+22.8 kJ mol^–1^) (Fig. 3b). Either form of **2′** was predicted to exist at higher energies than *M*_4_-**1′** (–58.1 kJ mol^–1^ relative to the most minimized potential energy for *M*_2_*P*_2_-**2′**).

Next, we investigated the dynamic structure in solution by NMR spectroscopy (Fig. S2†). The ^1^H NMR spectra of both **1** (**A**) and **2** (**B**) showed a single set of averaged resonances at room temperature (Fig. S2a†). The aromatic protons H^A^ on the central benzene ring of TPEB in **1** and **2** with two planes appeared more upfield compared to that in single-layer **7**, which might be characteristic of these two cyclophanes. The chemical shifts for H^B^ and H^D^ on the peripheral phenylethynyl blade of TPEB in **1** and **2** were close to those in macrocyclic **8** rather than those in **7**. This similarity indicated that macrocyclic **8** represented a substructure of these cyclophanes better than **7**. Energy-minimized structures for a model **8′** [NMe] are summarized in Fig. S3.† Since the chemical shifts for the averaged resonances in the spectra of **1** and **2** changed with temperature (Fig. S4a†), conformations with different structures underwent dynamic interconversions in each solution.^16^

The ^1^H NMR spectrum of (*R*)_4_-**3** (**A**) showed a single set of averaged resonances that included two differentiated singlet peaks for H^A^ and H^A′^, which indicated that global helical forms *M*_4_-**A** and *P*_4_-**A**, rather than an eclipsed form, predominated in solution, and interconverted to each other on the NMR timescale. Helical twisting of two planes in cyclophane **A** creates two non-equivalent spaces with different dimensions, where one is narrower than the other (Scheme 3a). In fact, several pairs on each of the upper and lower planes were differentiated (Fig. S2a†). Such differentiation due to a conformational preference for dynamic helical forms was also supported by ^13^C NMR (Fig. S2b†). If we consider that an eclipsed form is dominant, the two planes should be equivalent and should show a spectral pattern similar to that of single-layer **7**.

The ^1^H NMR spectra of both (*R*)_4_-**4** (**B**) and (*R*)_4_-**5** (**B**) showed a single set of averaged resonances with a spectral pattern similar to that of **7**, which could be explained with either form *M*_4_-**B**, *M*_2_*P*_2_-**B** or *P*_4_-**B**, and indicated that these diastereomeric forms undergo dynamic interconversions in solution.

### Control of screw-sense preference of dynamic helicity through the intramolecular transmission of central chirality (*R*) in (*R*)_4_-**3** (**A**), and through the arrangement of two different substitution groups in (*R*)_4_(*S*_p_)-**4** and (*R*)_4_(*R*_p_)-**5**

**Fig. 4:**
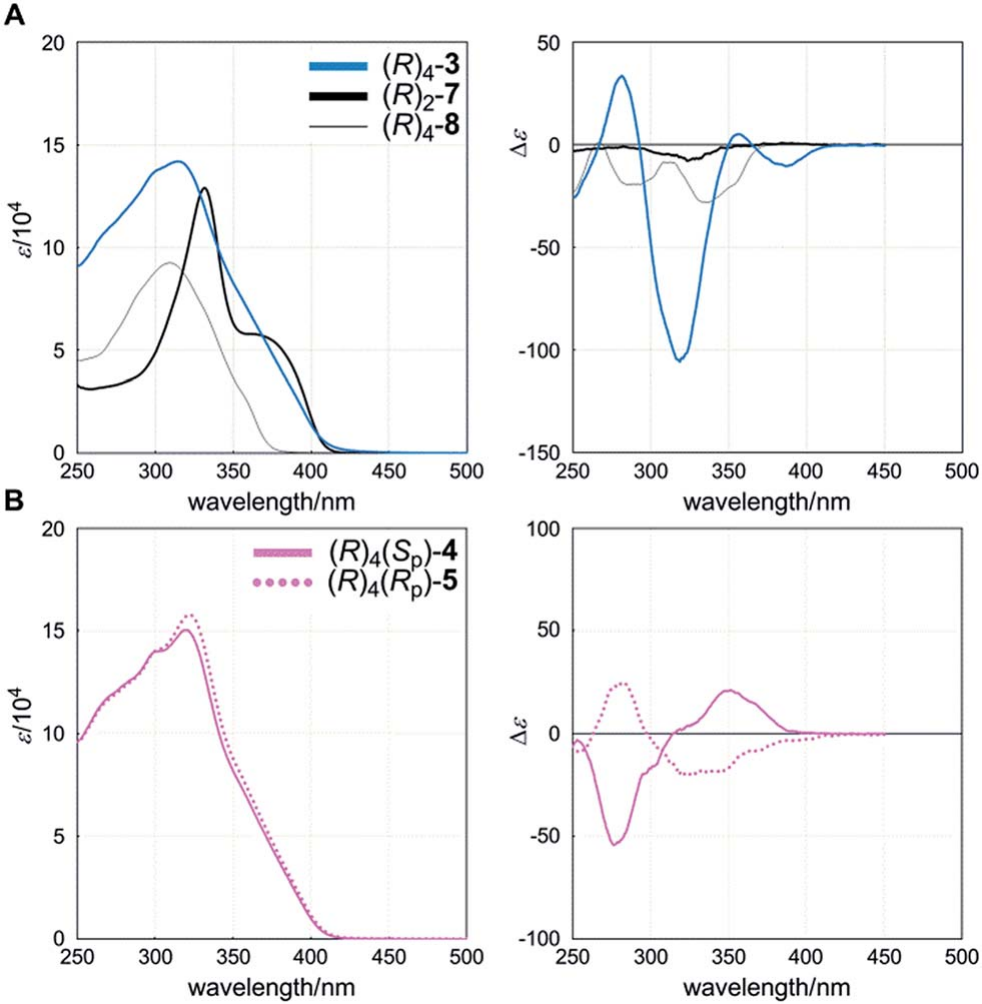
UV (left) and CD (right) spectra of (**A**) (*R*)_4_-**3**, (*R*)_2_-**7** and (*R*)_4_-**8**, and (**B**) (*R*)_4_(*S*_p_)-**4** and (*R*)_4_(*R*_p_)-**5**. All spectra were measured in dichloromethane at room temperature.

The UV-vis spectrum of a cyclophane (*R*)_4_-**3** (**A**) showed an absorption maximum [*λ*_max_/nm (log *ε*) 315 (5.15)] and a shoulder at a longer wavelength region (Fig. 4A, left), which seemed to be characteristic of TPEB,^17^ although they were hypsochromically shifted and the intensity was markedly attenuated throughout the absorption region, compared to the spectrum of single-layer TPEB **7** [332 (5.11) and sh. 375 (4.74)]. These spectral perturbations seen for **3** might be attributed to the reduction of coplanarity due to the local twisting of peripheral phenylethynyl blades with respect to the central benzene ring of TPEB. Instead, we found a similar appearance in the spectrum of macrocyclic **8** [309 (4.97)], which is composed of two chromophores of 1,4-bis(phenylethynyl)benzene, bridged by a two-fold terephthalamide, and such chromophores might be present as an effective conjugation even in **3**. In the CD spectrum of (*R*)_4_-**3** (**A**), we found compositive Cotton effects in the absorption region of **3** (Fig. 4A, right). On the other hand, in the spectrum of (*R*)_2_-**7**, small negatively signed Cotton effects were present throughout the absorption region of **7**. These Cotton effects of (*R*)_2_-**7** were completely different from those of (*R*)_4_-**3** (**A**), and were considered to have no relation with any helical structure, but rather originated from the local chiral environment around the central chiral auxiliary. Again, we found negatively signed Cotton effects in the spectrum of (*R*)_4_-**8**, although it was predicted to adopt helical forms. We considered that these negatively signed Cotton effects of (*R*)_4_-**8** could also be attributed to the local chiral environment, and that the intramolecular transmission of chirality would not be valid in a case where the two amide carbonyls in the bridging unit adopted a locally non-helical form (Fig. S3†).^18^ The Cotton effects seen for (*R*)_4_-**3** (**A**) could not be explained at all by assuming an eclipsed form or a local chiral environment around the chiral auxiliary, but could be explained by an induced preference for a particular screw sense of dynamic helicity through the intramolecular transmission of central chirality. At least one of the two chiral auxiliaries on the bridging unit should always be placed in a narrower space that is created by the helical twisting of the two planes in the cyclophane, and can act as a directing group to prefer a particular screw sense of dynamic helicity. We confirmed that the Cotton effects were enhanced with a decrease in temperature and attenuated with an increase in temperature (Fig. S6a†). This result indicated that the two diastereomeric forms with global (*M*)- or (*P*)-helicity undergo dynamic interconversion in solution.

Cyclophanes (*R*)_4_(*S*_p_)-**4** (**B**) and (*R*)_4_(*R*_p_)-**5** (**B**) showed absorption [*λ*_max_/nm (log *ε*) 320 (5.18) for **4**, and 322 (5.20) for **5**] similar in appearance to that of (*R*)_4_-**3** (**A**) (Fig. 4B, left). In these absorption regions, we found compositive and global bisignated Cotton effects in the CD spectra of each planar chiral cyclophane (Fig. 4B, right). Notably, the two spectra were pseudo-mirrored. These Cotton effects should be attributed to dynamic helical forms, rather than to the local chiral environment, since the identical chiral auxiliary was present in both cyclophanes. We considered that it may be difficult for a *meso*-like form to produce Cotton effects due to the intramolecular cancellation of partial helicity, even though it would be the most common form in solution. If the central chiral auxiliary preferred a particular screw sense of dynamic helicity through intramolecular transmission, it should be manifested because both dynamic helical forms *M*_4_-**B** and *P*_4_-**B** were provided in each planar chiral isomer (Scheme 4).^19^ If this assumption is valid, then similarly signed Cotton effects, not pseudo-mirrored, should appear in each spectrum. Thus, we considered that these Cotton effects showed an induced preference for a particular screw sense of dynamic helicity through the arrangement of two different substitution groups X and Y. VT CD measurements supported the contribution of diastereomeric forms with (*M*)- or (*P*)-helicity that dynamically interconverted in solution to the creation of pseudo-mirrored Cotton effects (Fig. S6b and c†).

In the following section, we confirmed the presence of dynamic helical forms that were unique to each type of cyclophane **A** and **B** using simple scaffolds **1** (**A**) and **2** (**B**), which do not possess any chiral element other than dynamic helicity (Scheme 3).

### Control of screw-sense preference of dynamic helicity through the supramolecular transmission of central chirality (*S*,*S*) or (*R*,*R*) in the guest to dynamic helicity of achiral cyclophane hosts **1** (**A**) and **2** (**B**)

**Fig. 5:**
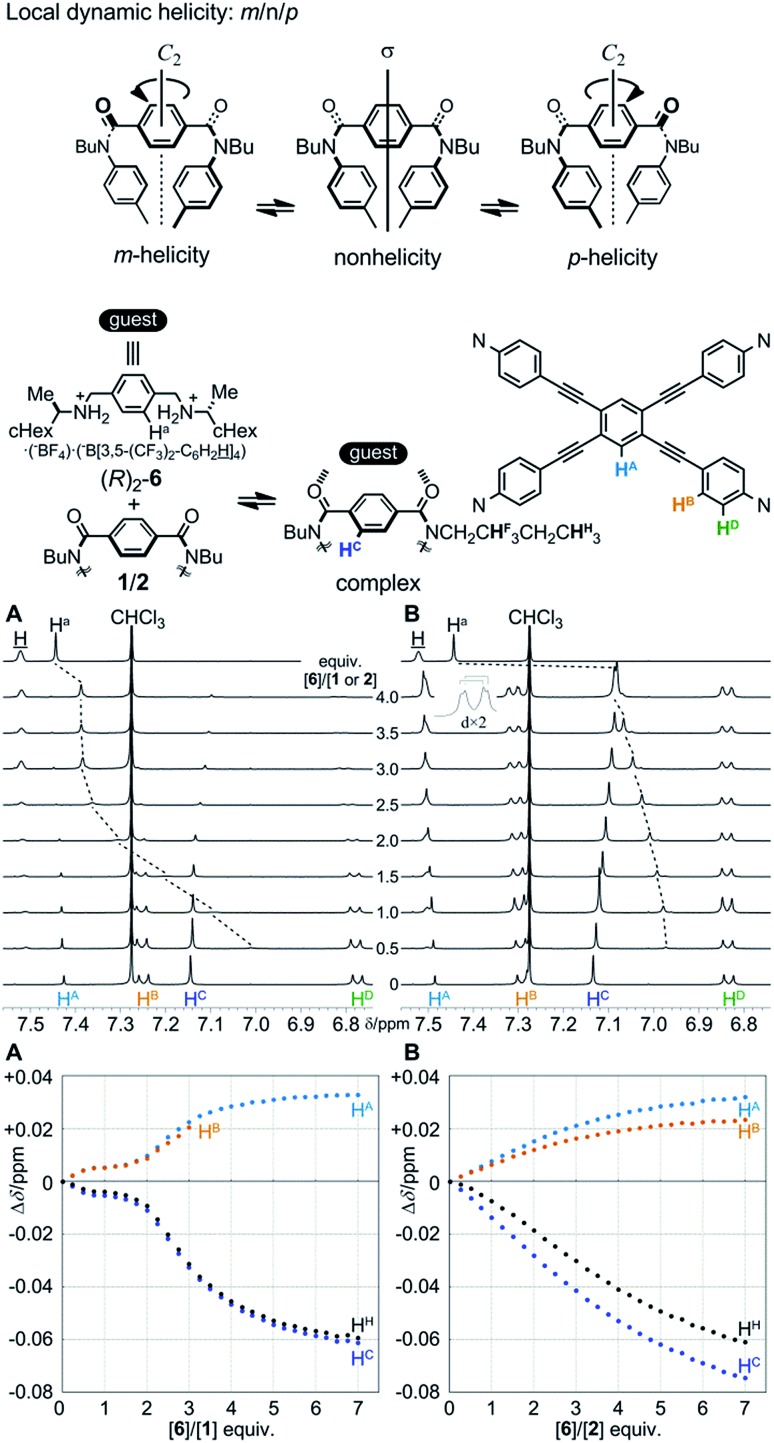
Partial ^1^H NMR (400 MHz) spectra of **1** (**A**) (0.5 mM)/**2** (**B**) (0.5 mM) in the presence of (*R*)_2_-**6** [0 (host only)-4 equiv.] and the spectrum of (*R*)_2_-**6** (top), and titration curves for the complexation of **1** (**A**)/**2** (**B**) with (*R*)_2_-**6**. All spectra were measured in 2 vol% acetonitrile-*d*_3_/chloroform-*d* at 298 K.

Two amide carbonyls in the bridging unit are allowed to twist helically in a conrotatory (*m*- or *p*-helicity) or disrotatory manner (non-helicity) around a local *C*_2_ axis. Thus, we considered that helical twisting of the two amide carbonyls would provide local dynamic helicity in the bridge (Fig. 5). We envisioned that the control of local dynamic helicity would lead to global control of the preference for a dynamic helical form *M*_4_- or *P*_4_- in these cyclophanes [**1** (**A**) and **2** (**B**)]. The direction of the local twisting of the two amide carbonyls would be controlled by the supramolecular transmission of central chirality, when a chiral ditopic guest is captured at the two amide carbonyls.

We first investigated the complexation of cyclophane **1** (**A**) with a chiral ditopic guest (*R*)_2_-**6** by ^1^H NMR spectroscopy, measured in chloroform-*d* containing 2 vol% acetonitrile-*d*_3_ at 298 K (Fig. 5A). When the host and guest were mixed, we found complexation-induced shifts for both phenylene protons H^C^ and H^a^, associated with **1** and **6**, respectively, which indicated that the guest was captured at the two amide carbonyls of a bridging unit through the formation of double hydrogen bonds. Through a titration experiment, we obtained complicated discontinuous titration curves that included several inflection points, especially at around the addition of two equivalents of **6**. In an early stage, we could confirm that the guest was mostly in a complexed state through ditopic binding, as shown by a large upfield shift for H^a^, which was later insignificant upon the further addition of **6**. For these inflection points, we could not analyze the complexation quantitatively. Although signals were broadened in a later stage, we tried to trace changes in the chemical shift for other protons far from the binding site, which indicated that complexation induced some change in the conformation of **1**.

Next, we investigated the complexation of **2** (**B**) with (*R*)_2_-**6** under similar conditions (Fig. 5B). Similar to the above case, we confirmed complexation-induced shifts for both phenylene protons H^C^ and H^a^, which indicated the formation of double hydrogen bonds at the two amide carbonyls. Unlike the above case, we fortunately obtained titration curves that showed a sigmoidal curve, which indicated that we could analyze complexation as a type of positive allosteric binding.8e–g,^20^ In a later stage, we found that aromatic protons H^B^ (and H^D^) on TPEB in **2** (**B**) were differentiated, as seen for **4** (**B**) or **5** (**B**). Although no conformation of the host is homotopic (Scheme 3b), we tried to analyze the complexation by a Job plot and Hill plot (Fig. S8†).^21^ In Job plots, we could find a maximum or minimum for several protons throughout the host molecule at 0.2 < *χ***_2_** < 0.3, and for protons in the guest at 0.7 < *χ*_**6**_ < 0.8 (Fig. S8a†). We estimated that the binding constant *K*_a_ was 10^8^ to 10^9^ M^–4^ and the Hill coefficient was 2.5–2.7 on the basis of Hill plots for several protons (Fig. S8b†), if we assumed that a 1 : 4 complex was formed.

**Fig. 6:**
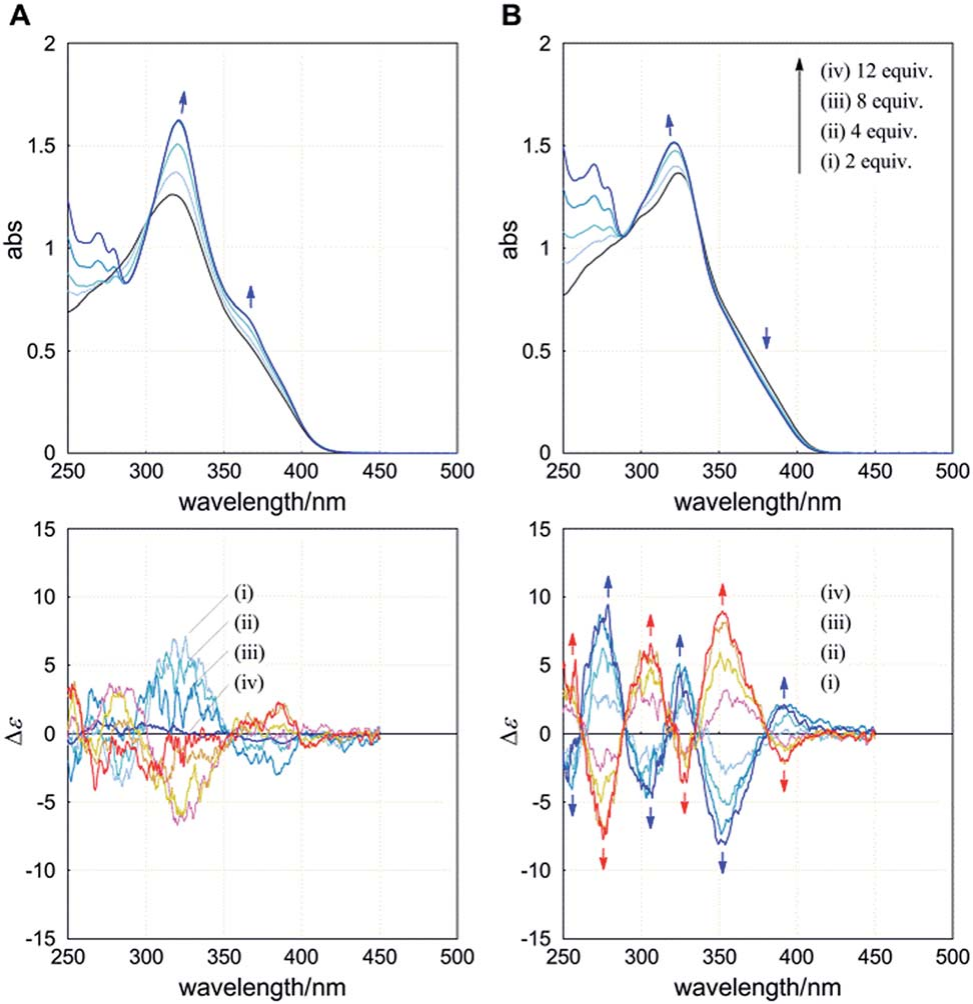
UV (upper) and CD (lower) spectra of **1** (**A**) (7.5 × 10^–5^ M) and **2** (**B**) (9.9 × 10^–5^ M) in the presence of (*R*)_2_-**6** (blue lines) or (*S*)_2_-**6** (red lines) [0 (black, host only), 2, 4, 8 and 12 equiv.]. All spectra were measured in dichloromethane at 293 K.

The UV-vis spectrum of **1** (**A**) showed an absorption maximum at 318 nm and a shoulder band at around 360 nm. When we added (*R*)_2_-**6** to a solution of **1** in dichloromethane at room temperature, the former band increased with a slight bathochromic shift and the latter band increased (Fig. 6A, upper), which seemed to change toward the spectrum of **7**. In an early stage, small but significant Cotton effects similar to those in the spectrum of (*R*)_4_-**3** (**A**) were induced in the absorption region of **1** (Fig. 6A, lower). Addition of the antipodal guest (*S*)_2_-**6** induced mirror-imaged Cotton effects. These results indicated that a particular screw sense of dynamic helicity was preferred in a complex, at least in an early stage. These Cotton effects were attenuated and ultimately disappeared upon further addition of the guest. We considered that these spectral changes resulted from the cyclophane host **1** undergoing a change in conformation from dynamic helical forms to a less- or non-helical form during complexation.

The UV-vis spectrum of **2** (**B**) showed an absorption maximum at 324 nm and a shoulder band at a longer wavelength (Fig. 6B, upper). When we added (*R*)_2_-**6** to a solution of **2** under conditions similar to those in the above case for **1**, the former band increased with a slight hypsochromic shift and the latter band decreased. In the CD spectrum of **2**, we found a gradual increase in the Cotton effects to show several compositive couplets upon gradual addition of the guest (Fig. 6B, lower). When the antipodal guest (*S*)_2_-**6** was added, completely mirror-imaged Cotton effects were induced. These spectral changes indicated a complexation-induced change in conformation from an inherently twisted but *meso*-like form *M*_2_*P*_2_ to dynamic helical forms *M*_4_ or *P*_4_, and the cyclophane host preferred a particular screw sense of dynamic helicity generated in a complex through the supramolecular transmission of central chirality in the guest.

## Conclusions

We have demonstrated the design and control of screw-sense preference of unique dynamic helicity that was generated by the twisting of two planes in a cyclophane (dynamic helical cyclophane). We used a rectangular and anisotropic plane that was stacked in parallel or orthogonally in pairs in two distinct cyclophanes with *D*_2h_ (**A**) or *D*_2d_ (**B**) symmetry. We considered that such two planes in each cyclophane are in an “obverse and/or reverse” relation. The arrangement of two different substitution groups X and Y (X ≠ Y) on bridges generates planar chirality in a particular cyclophane of type **A**^13^ or **B**. These planar chiral cyclophanes are configurationally stable and conformationally dynamic. We confirmed that the two different substitution groups can act as directing groups to control the screw-sense preference of unique dynamic helicity in each planar chiral cyclophane through the arrangement of two different substitutions, independent of any transmission of chirality.

## Supplementary Material

Supplementary information

Crystal structure data
