# Supplementary material for: Dynamic helical cyclophanes with two quadruply-bridged planes arranged in an “obverse and/or reverse” relation
Source: Chem Sci. 2016 Jan 29;7(5):3240–7. doi: 10.1039/c5sc04673d (PMC6006470; doi:10.1039/c5sc04673d)
Supplement: Supplementary file 1 [file SC-007-C5SC04673D-s001.pdf]

Electronic Supplementary Material (ESI) for Chemical Science.
This journal is © The Royal Society of Chemistry 2016

Supplementary Information

Dynamic helical cyclophanes with two quadruply-bridged planes arranged in an "obverse and/or reverse" relation

Ryo Katoono,* Shunsuke Kawai and Takanori Suzuki

Department of Chemistry, Faculty of Science, Hokkaido University

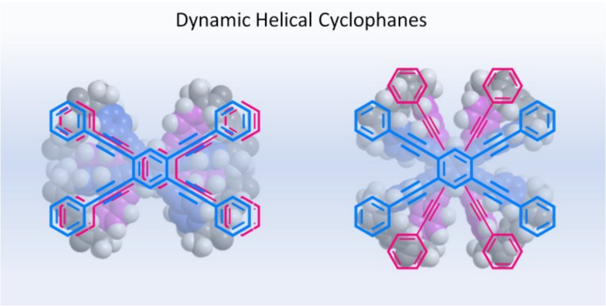

Contents

Supplementary Figures (**Fig. S1-Fig. S8**)	S2-S8
Experimental (**Scheme S1** and **Scheme S2**)	S9-17
References	S17

Supplementary Figures

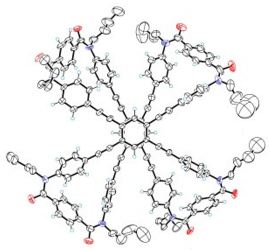

Fig. S1 X-ray structure of **2**. Solvents are omitted for clarity. Due to the large thermal motion of the butyl groups, the hydrogen atoms on the butyl carbons are not included in the structural refinement.

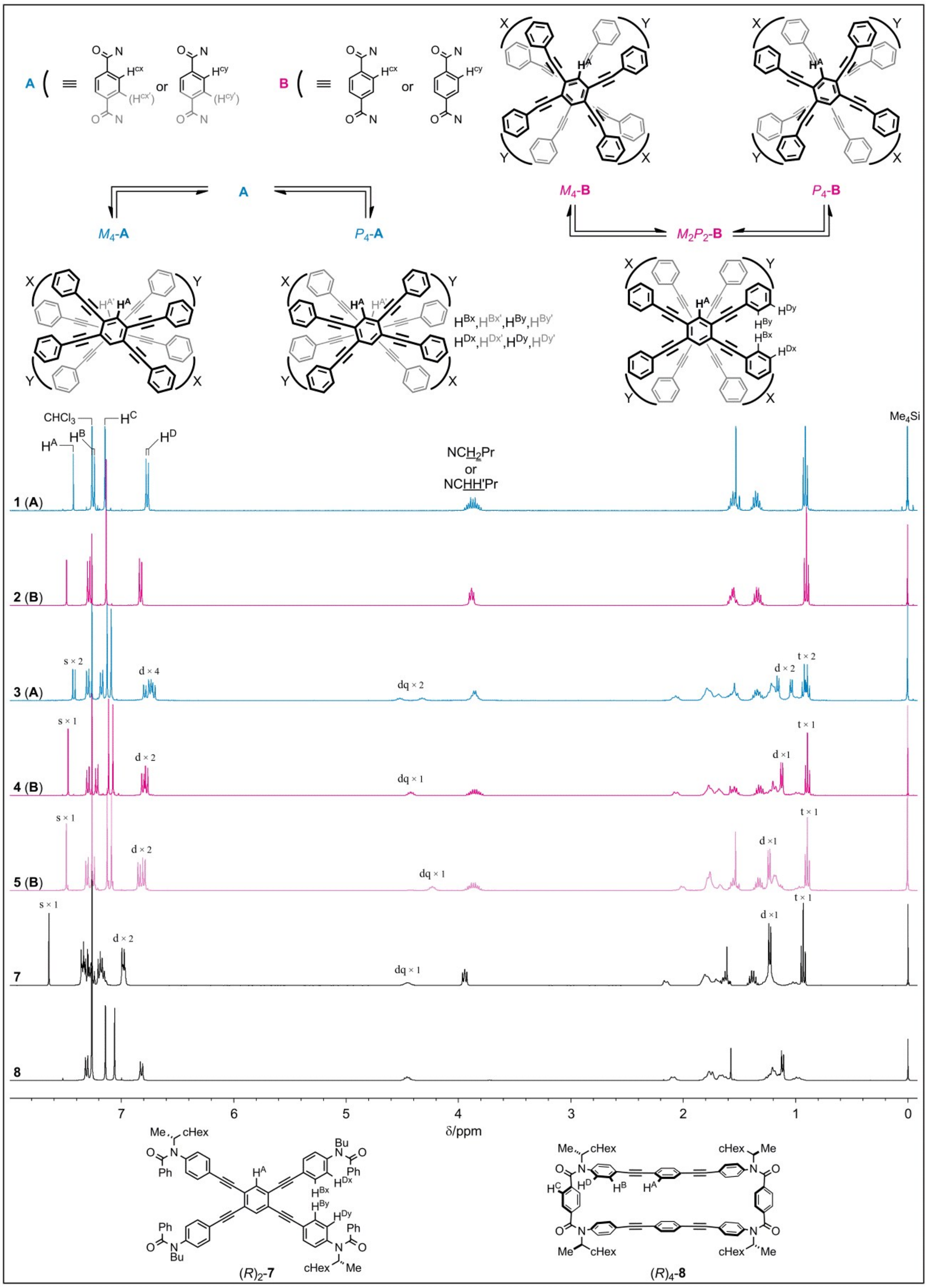

Fig. S2a ^{1}H NMR (400 MHz) spectra of **1**, **2**, (R)$_4$-**3**, (R)$_4$(S_p)-**4**, (R)$_4$(R_p)-**5**, (R)$_2$-**7** and (R)$_4$-**8**, measured in chloroform-*d* at room temperature. As a representative planar chiral diastereomer (**B**), only an (R_p)-isomer is illustrated.

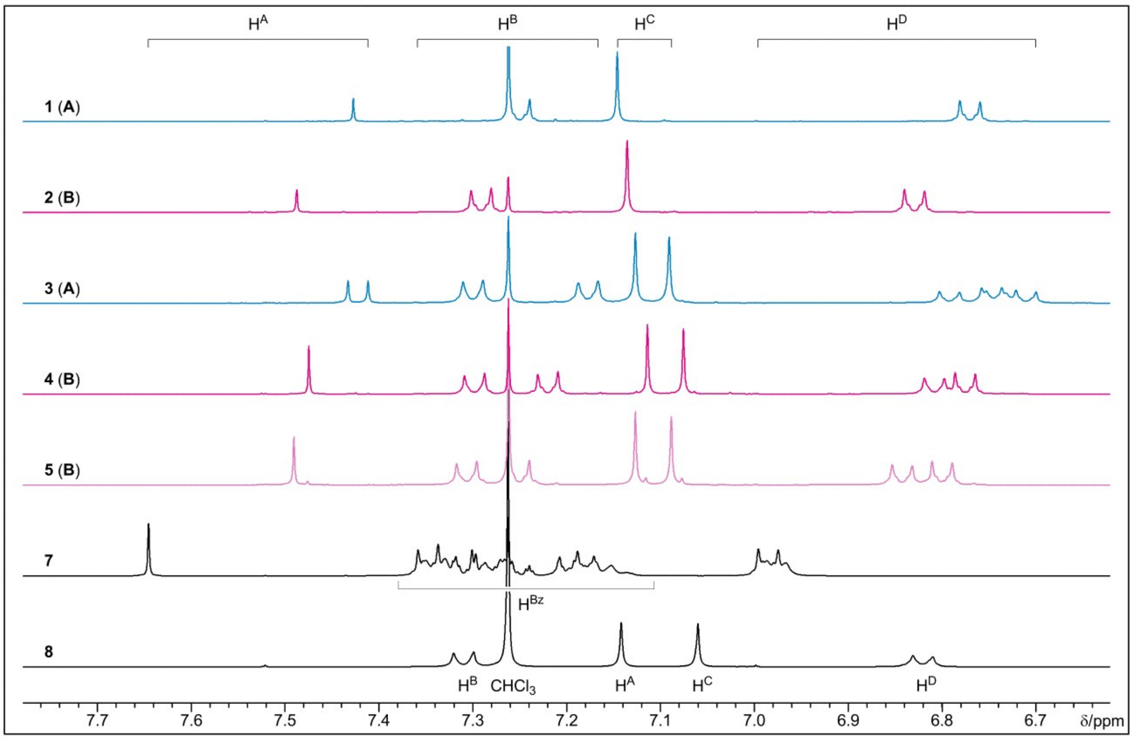

The aromatic region of the ^{1}H NMR (400 MHz) spectra of **1**, **2**, (R)$_4$-**3**, (R)$_4$(S_p)-**4**, (R)$_4$(R_p)-**5**, (R)$_2$-**7** and (R)$_4$-**8**, measured in chloroform-*d* at room temperature.

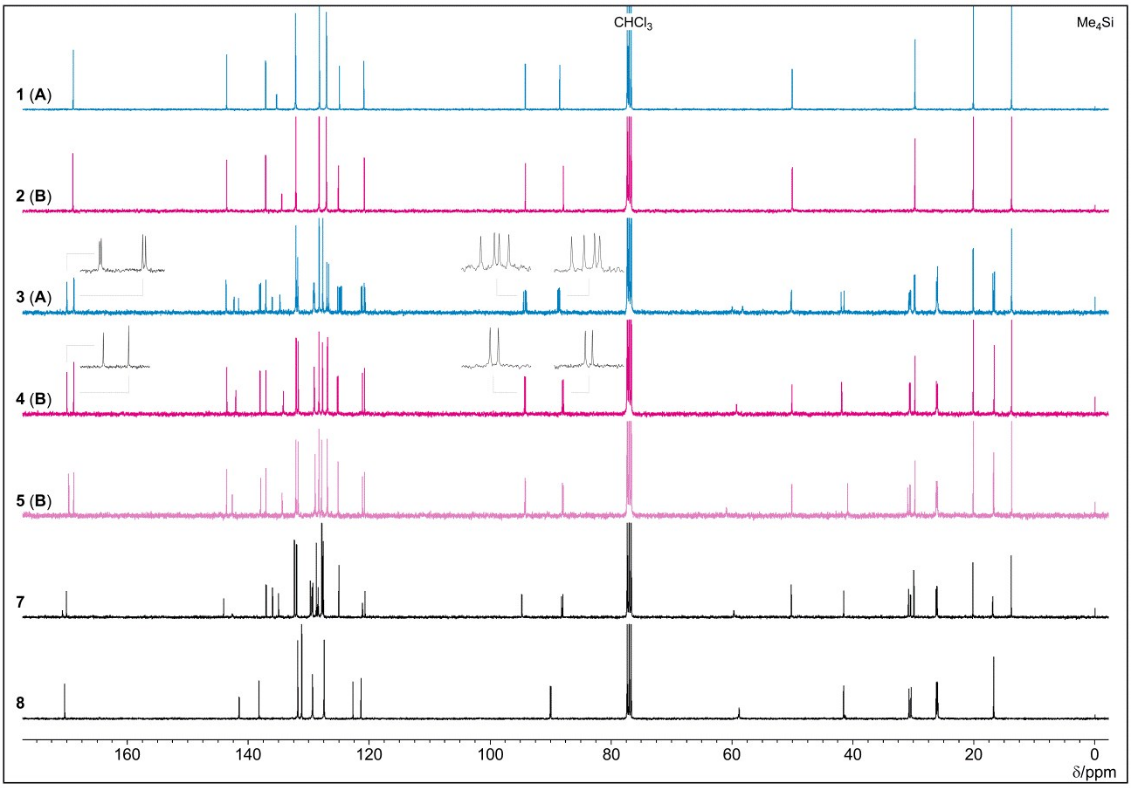

Fig. S2b ^{13}C NMR (100 MHz) spectra of **1**, **2**, (R)$_4$-**3**, (R)$_4$(S_p)-**4**, (R)$_4$(R_p)-**5**, (R)$_2$-**7** and (R)$_4$-**8**, measured in chloroform-*d* at room temperature.

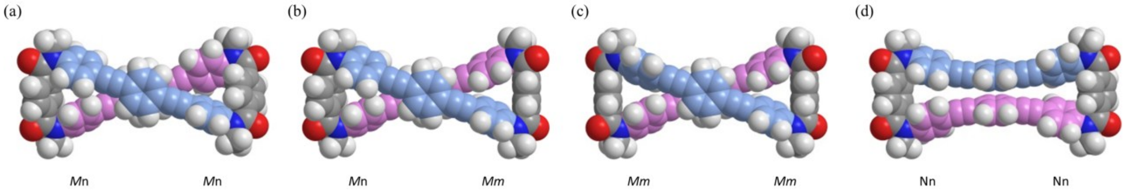

Fig. S3 Energy-minimized structures for a model **8'** [NMe] (a) (*M*n)$_2$ (rel. 0 kJ mol^{-1}), (b) (*M*n)(*Mm*) (+2.66 kJ mol^{-1}), (c) (*Mm*)$_2$ (+5.35 kJ mol^{-1}) and (d) (Nn)$_2$ (+17.2 kJ mol^{-1}), obtained by a conformational search using MacroModel software (v9.9 OPLS_2005, Monte Carlo Multiple Minimum method, non-solvated, 20 000 steps). With regard to the local conformations of the two amide carbonyls in the bridging unit, we denote a non-helical form as lowercase n and dynamic helical forms as lowercase *m* and *p*, in addition to the global helicity.

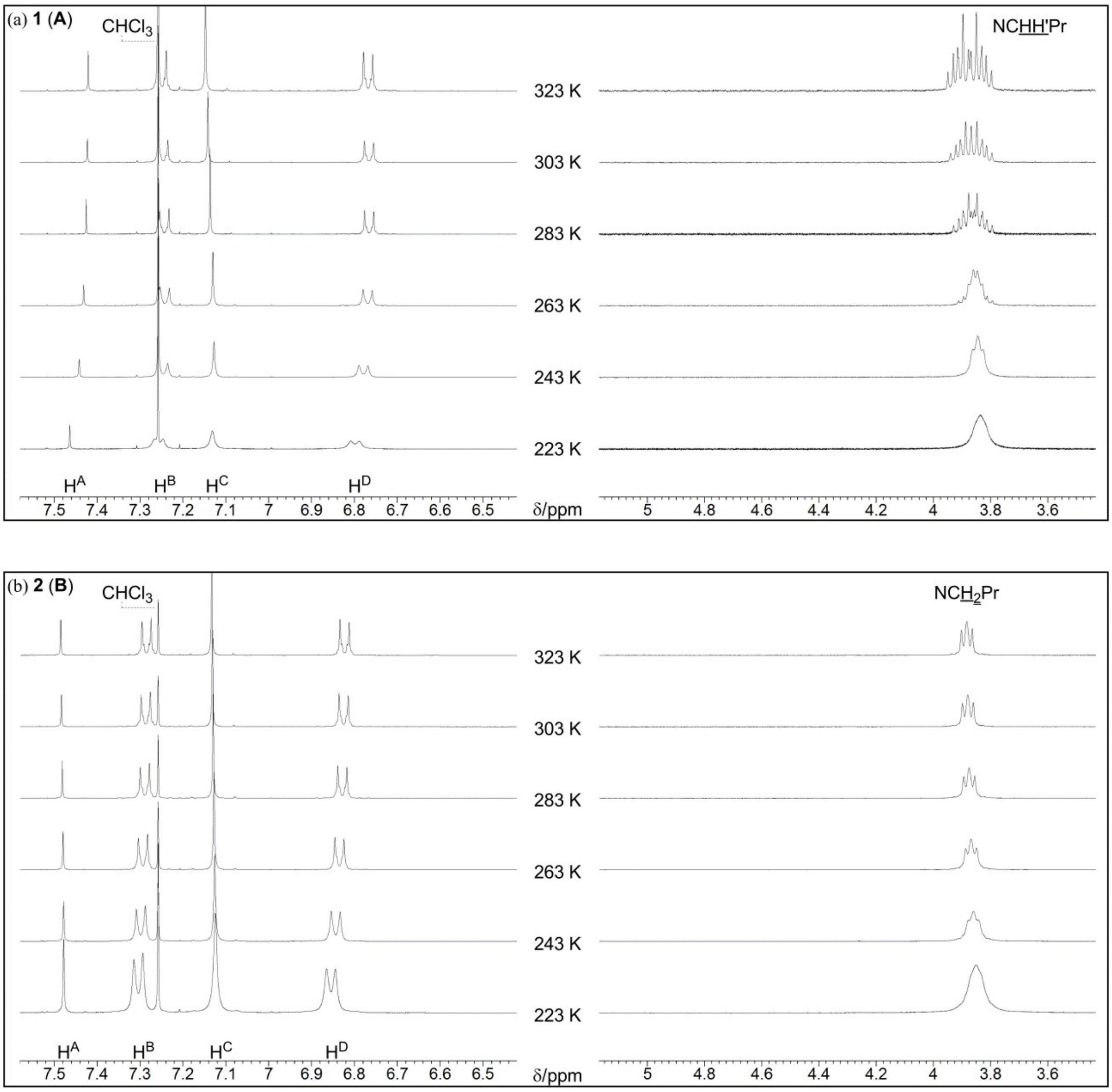

Fig. S4a VT ^{1}H NMR (400 MHz) spectra of (a) **1** and (b) **2**, measured in chloroform-*d* at 223-323 K.

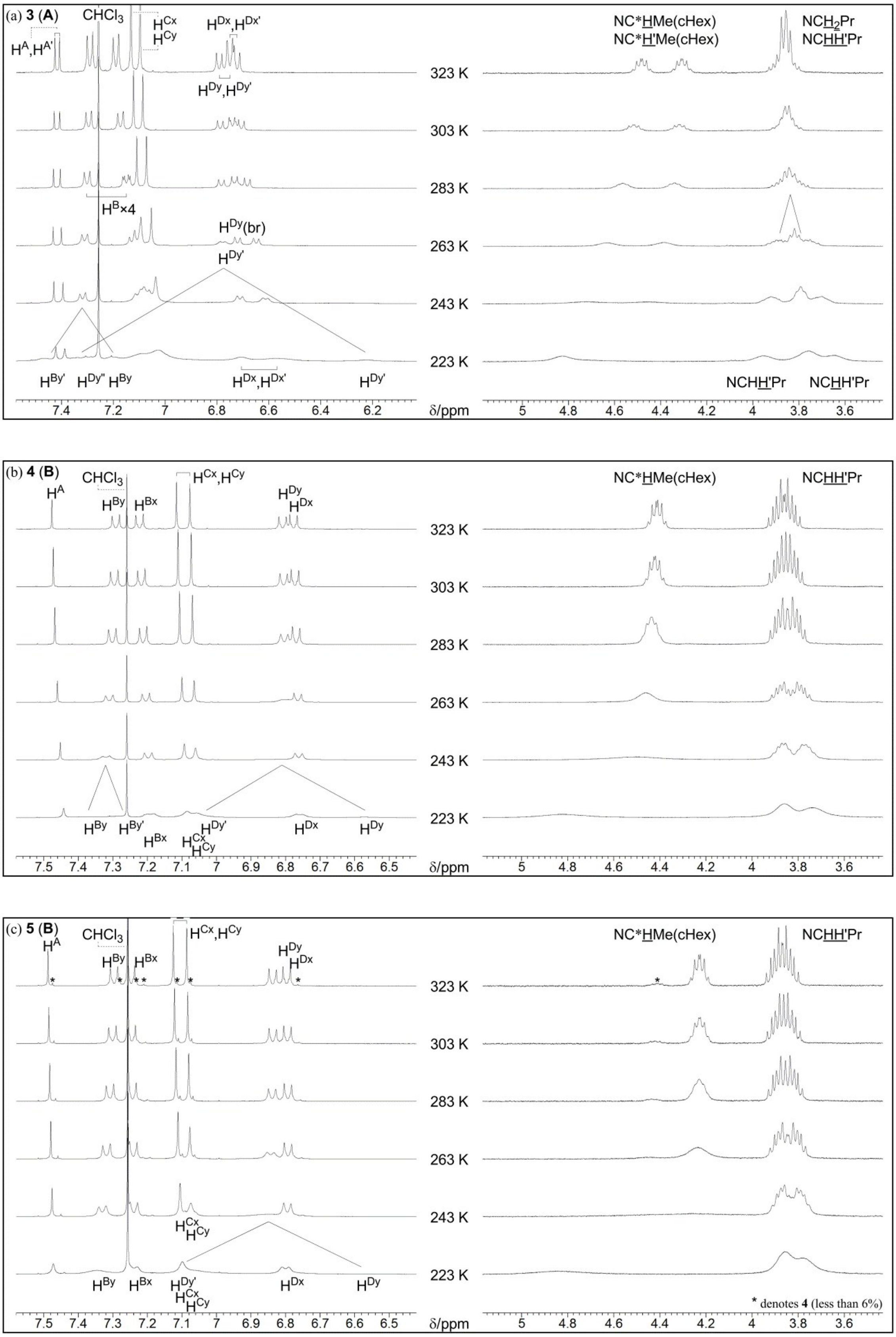

Fig. S4b VT ^{1}H NMR (400 MHz) spectra of (a) $(R)_4$-**3**, (b) $(R)_4(S_p)$-**4** and (c) $(R)_4(R_p)$-**5**, measured in chloroform-*d* at 223-323 K.

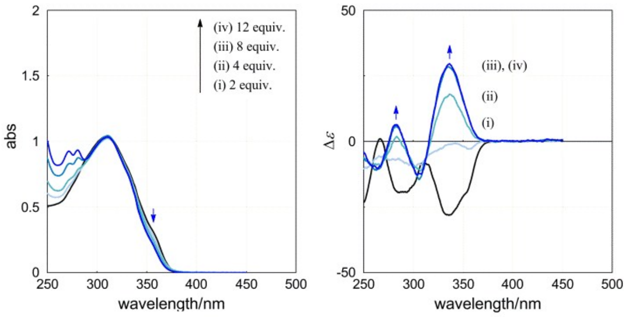

Fig. S5 Changes in the UV (left) and CD (right) spectra of $(R)_4$-**8** (1.1×10^{-4} M) in the presence of $(R)_2$-**6** (blue lines) [0 (black line, host only), 1, 2, 4 and 6 equiv.]. All spectra were measured in dichloromethane at room temperature.

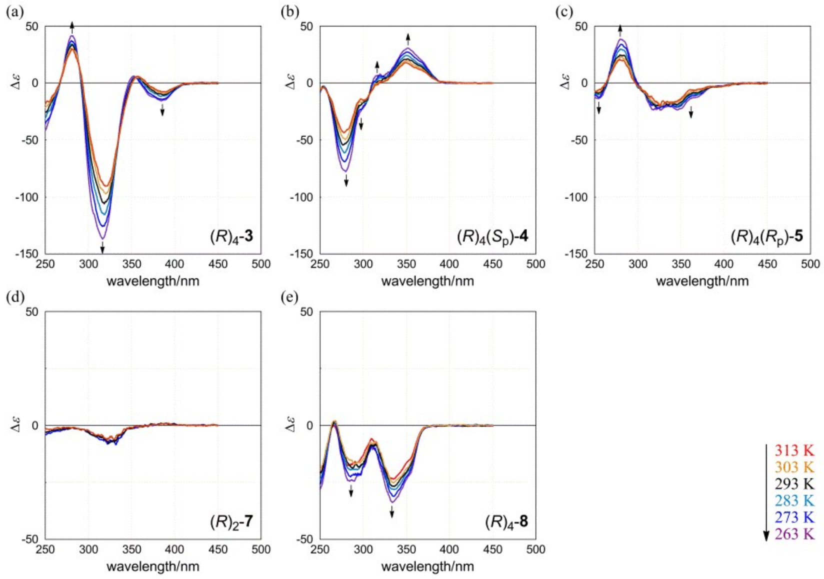

Fig. S6 VT CD spectra of (a) $(R)_4$-**3**, (b) $(R)_4(S_p)$-**4**, (c) $(R)_4(R_p)$-**5**, (d) $(R)_2$-**7** and $(R)_4$-**8**, measured in dichloromethane at 263-313 K.

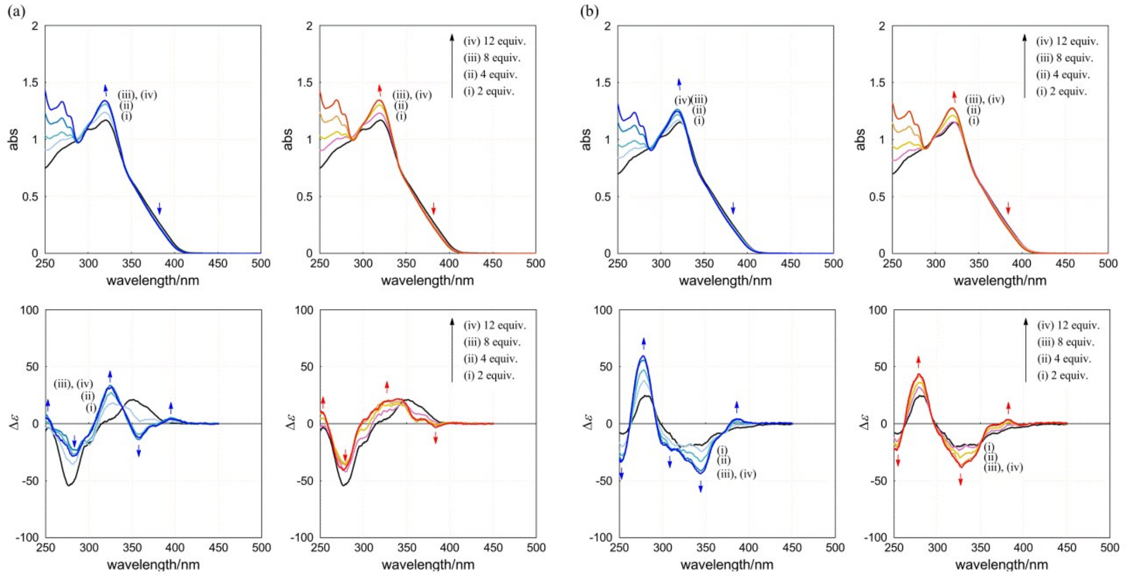

Fig. S7 Changes in the UV (upper) and CD (lower) spectra of (a) $(R)_4(S_p)$-**4** (**B**) (7.8×10^{-5} M) and (b) $(R)_4(R_p)$-**5** (**B**) (7.3×10^{-5} M) upon complexation with $(R)_2$-**6** (blue lines) or $(S)_2$-**6** (red lines) [0 (black line, host only), 2, 4, 8 and 12 equiv.]. All spectra were measured in dichloromethane at room temperature.

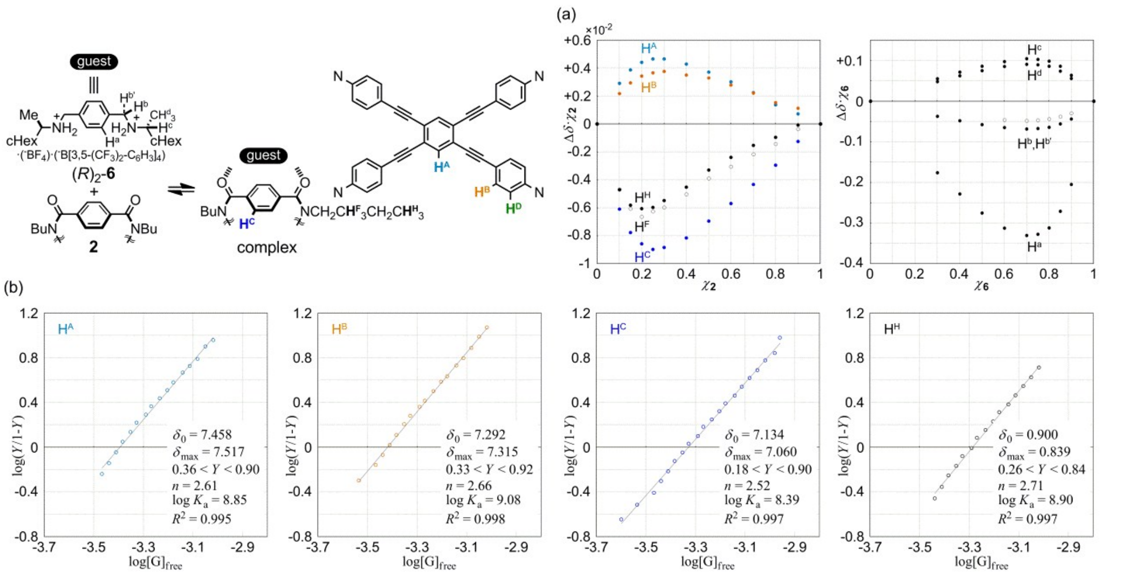

Fig. S8 (a) Job plot ([**2**] + [**6**] = 2 mM, left for **2** and right for **6**) and (b) Hill plot ([**2**] = 0.5 mM, [**6**] = 0-12 mM) for the complexation of **2** (**B**) with $(R)_2$-**6** in 2vol% acetonitrile-d_3/chloroform-d at 298 K.

Experimental

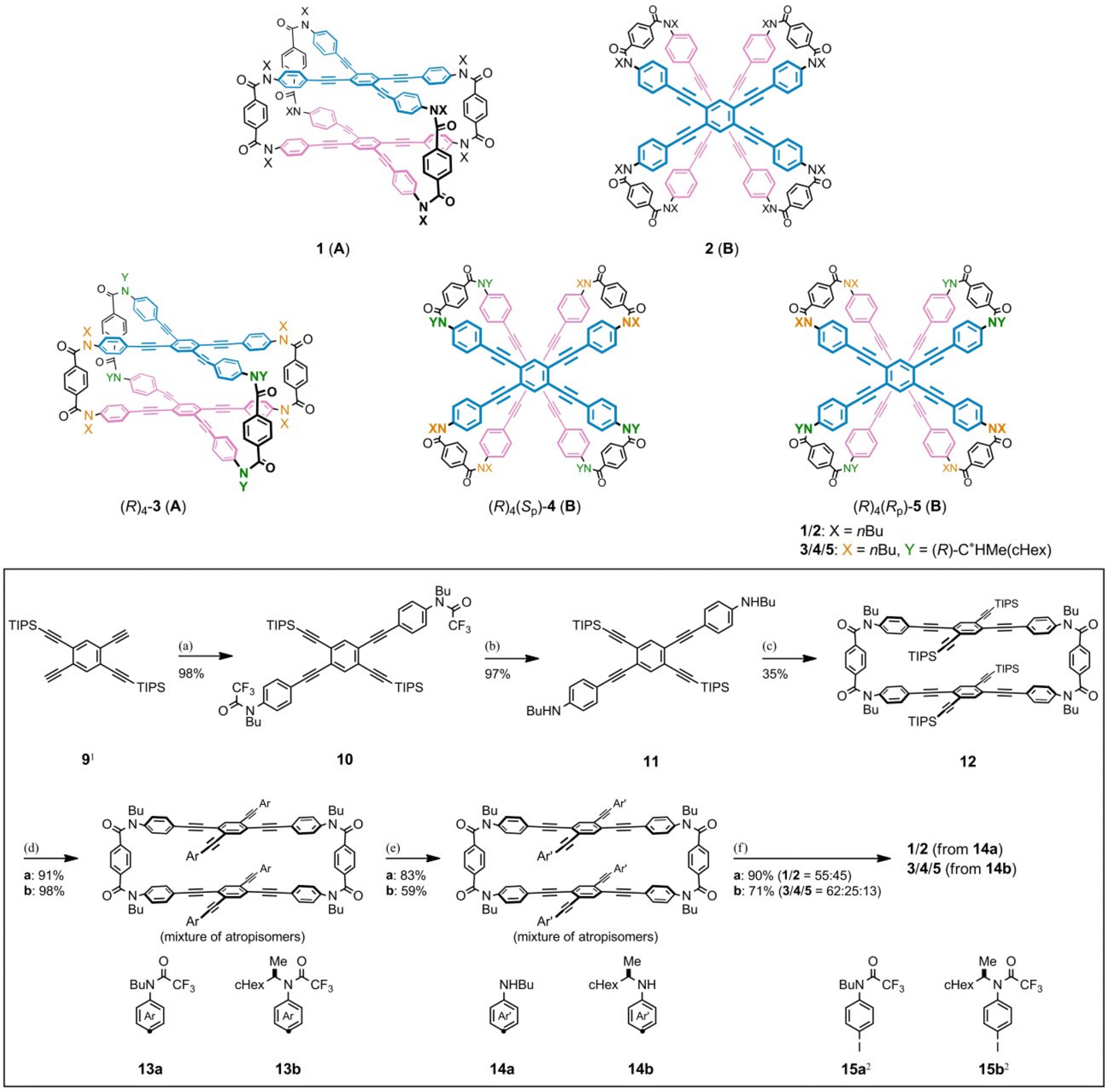

Scheme S1. Preparation of cyclophanes **1**[3]/**2** and **3-5**. Reagents: (a) **15a**,[2] $Pd(PPh_3)_4$, CuI, $i$$Pr_2NH$, THF; (b) NaH, MeOH, THF; (c) terephthaloyl chloride, Et_3N, toluene, THF; (d) **15a**/**b**,[2] $Pd(PPh_3)_4$, CuI, TBAF, Et_3N, THF; (e) NaH, MeOH, THF; (f) terephthaloyl chloride, Et_3N, toluene, THF.

Preparation of **10**

To a solution of **9**[1] (1.13 g, 2.33 mmol) and **15a**[2] (2.60 g, 7.02 mmol) in THF (12 mL) and $i$$Pr_2NH$ (12 mL) were added $Pd(PPh_3)_4$ (162 mg, 0.140 mmol) and CuI (55 mg, 0.29 mmol) at room temperature under an argon atmosphere, and the mixture was stirred at 54 ºC for 19 hours. After removal of a solid by filtration, the filtrate was concentrated and purified by column chromatography on SiO_2 (dichloromethane/hexane) to give **10** (2.22 g) as a white solid in 98% yield. An analytical sample was obtained as colorless crystals by recrystallization from ethanol. **10**: mp 166-167 °C; elemental analyses Found: C, 69.09; H, 7.34; N, 2.86%. Calc. for $C_{56}H_{70}F_6N_2O_2$: C, 69.10; H, 7.25; N, 2.88%; IR (KBr) ν_{max}/cm^{-1} 2941, 2865, 2222, 2151, 1698, 1508; ^{1}H NMR δ_H(400 MHz; $CDCl_3$;

Me_4Si)/ppm 7.67 (2H, s), 7.58 (4H, d, J = 8.4 Hz), 7.20 (4H, d, J = 8.4 Hz), 3.75 (4H, t, J = 7.6 Hz), 1.59-1.51 (4H, m), 1.35 (4H, sext, J = 7.6 Hz), 1.15-1.07 (42H, m), 0.92 (6H, t, J = 7.2 Hz); ^{13}C NMR δ_C(100 MHz; $CDCl_3$)/ppm 156.5 ($\underline{C}$(=O)CF_3), 139.1, 136.1, 132.7, 128.3, 125.5, 125.1, 123.8, 116.4 ($\underline{C}F_3$), 103.9, 98.1, 93.5, 88.9, 51.6, 28.9, 19.8, 18.6, 13.7, 11.3; FD-LRMS *m*/*z* 972.51 (M^+, 100%), 973.52 ($[M+1]^+$, 74), 974.52 ($[M+2]^+$, 33), 975.52 ($[M+3]^+$, 12).

Preparation of **12**

To an ice-cooled solution of **10** (1.24 g, 1.27 mmol) in THF (35 mL) were added 60% NaH in oil (137 mg, 3.43 mmol) and MeOH (1.1 mL), and the mixture was stirred at room temperature for 10 mim, and then diluted with dichloromethane. The organic layer was washed with aq. $NaHCO_3$, dried over magnesium sulfate, and then purified by column chromatography on SiO_2 (dichloromethane/hexane) to give **11** (968 mg) as a bright yellow solid in 97% yield, which was immediately subjected to the next reaction.

To a solution of terephthaloyl chloride (557 mg, 2.74 mmol) in toluene (200 mL) and THF (100 mL) were added 10 mL of a solution of **11** (1.96 g, 2.51 mmol) in THF (18 mL) containing Et_3N (1.8 mL, 13 mmol) at room temperature over a period of 1 hour, and then additional THF (80 mL). To the diluted mixture were added the rest of the solution containing **11** and additional THF (50 mL) at 93 ºC, and the reaction mixture was stirred at 100 ºC for 40 min. After removal of a solid by filtration, the filtrate was concentrated. The residue was dissolved in dichloromethane, which was washed with 2M aq. NaOH, dried over magnesium sulfate, and then purified by column chromatography on SiO_2 (ethyl acetate/dichloromethane), followed by GPC (chloroform) to give **12** (807 mg) as a white solid in 35% yield. An analytical sample was suspended in refluxed acetone, and collected as a white solid. **12**: mp >300 °C; elemental analyses Found: C, 78.76; H, 8.13; N, 3.05%. Calc. for $C_{120}H_{148}N_4O_4Si_4$: C, 79.07; H, 8.18; N, 3.07%; IR (KBr) ν_{max}/cm^{-1} 3041, 2940, 2863, 2221, 2155, 1655, 1650, 1512; ^{1}H NMR δ_H(400 MHz; $CDCl_3$; Me_4Si)/ppm 7.32 (4H, s), 7.27 (8H, d, J = 8.4 Hz), 7.16 (8H, s), 6.79 (8H, d, J = 8.4 Hz), 3.89 (8H, br s), 1.57-1.49 (8H, m), 1.32 (8H, sext, J = 7.6 Hz), 1.21-1.11 (84H, m), 0.90 (12H, t, J = 7.6 Hz); ^{13}C NMR δ_C(100 MHz; $CDCl_3$)/ppm 169.0, 143.1, 137.1, 135.8, 132.3, 128.3, 126.8, 124.9, 124.8, 121.4, 104.4, 96.7, 93.9, 89.0, 50.0, 29.7, 20.1, 18.8, 13.8, 11.4; FD-LRMS *m*/*z* 1821.10 (M^+, 66%), 1822.11 ($[M+1]^+$, 100), 1823.11 ($[M+2]^+$, 89), 1824.11 ($[M+3]^+$, 55), 1825.12 ($[M+4]^+$, 28), 1826.12 ($[M+5]^+$, 11).

Preparation of **13a** (mixture of atropisomers) [X = *n*Bu]

To a solution of **12** (173 mg, 0.0949 mmol), **15a** (239 mg, 0.644 mmol), $Pd(PPh_3)_4$ (23 mg, 0.020 mmol) and CuI (10 mg, 0.053 mmol) in THF (5 mL) and Et_3N (14 mL) was added a solution of TBAF (0.40 mmol) in THF (2 mL) over a period of 2 hours via a syringe pump at 49-50 °C under an argon atmosphere, and the mixture was stirred at that temperature for 30 min. After removal of the solvents by evaporation, the residue was dissolved in ethyl acetate, which was washed with 0.1M

aq. HCl, and brine, dried over magnesium sulfate, and then purified by column chromatography on SiO_2 (ethyl acetate/dichloromethane) to give **13a** (187 mg) as a yellow solid in 91% yield (a mixture of atropisomers).

Preparation of **13b** (mixture of atropisomers) [X = *n*Bu, Y = (*R*)-CHMe(cHex)]

To a solution of **12** (758 mg, 0.416 mmol), **15b**[2] (1.03 g, 2.43 mmol), $Pd(PPh_3)_4$ (97 mg, 0.084 mmol) and CuI (18 mg, 0.094 mmol) in THF (27 mL) and Et_3N (60 mL) was added a solution of TBAF (1.76 mmol) in THF (8.4 mL) over a period of 2 hours via a syringe pump at 49-51 °C under an argon atmosphere, and the mixture was stirred at that temperature for 10 min. After removal of the solvents by evaporation, the residue was dissolved in ethyl acetate, which was washed with 0.1M aq. HCl, and brine, dried over magnesium sulfate, and then purified by column chromatography on SiO_2 (ethyl acetate/dichloromethane) to give **13b** (970 mg) as a yellow solid in 98% yield (a mixture of atropisomers).

Preparation of **1** and **2** [X = *n*Bu]

To an ice-cooled solution of **13a** (452 mg, 0.208 mmol) in THF (6 mL) were added 60% NaH in oil (47 mg, 1.2 mmol) and MeOH (0.4 mL), and the mixture was stirred at that temperature for 5 min, and then diluted with dichloromethane. The organic layer was washed with aq. $NaHCO_3$, dried over magnesium sulfate, and then purified by column chromatography on SiO_2 (ethyl acetate/dichloromethane) to give **14a** (310 mg) as a bright yellow solid in 83% yield, which was immediately subjected to the next reaction.

To a solution of **14a** (310 mg, 0.174 mmol) in toluene (70 mL) and THF (40 mL) containing Et_3N (0.25 mL, 1.8 mmol) was added terephthaloyl chloride (85 mg, 0.42 mmol), and the mixture was stirred at 80 °C for 35 min. After removal of a solid by filtration, the filtrate was concentrated. The residue was purified by column chromatography on SiO_2 (ethyl acetate/dichloromethane), followed by GPC (chloroform) to give a mixture of **1** and **2** (318 mg) as a yellow solid in 90% yield. HPLC separation (1:3 and 2:3 ethyl acetate/chloroform) gave **1**[3] and **2** in pure form, respectively. Each analytical sample was obtained as a pale yellow solid by reprecipitation in ethyl acetate with hexane.

2: mp 253-260 °C (dec); UV $\lambda_{max}(CH_2Cl_2)$/nm 324 (log ε 5.22); IR (KBr) ν_{max}/cm^{-1} 3040, 2955, 2929, 2870, 2204, 1650, 1600, 1513, 1378, 1294, 836, 725; ^1H NMR δ_H(400 MHz; $CDCl_3$; Me_4Si)/ppm 7.49 (4H, s), 7.29 (16H, d, *J* = 8.8 Hz), 7.14 (16H, s), 6.83 (16H, d, *J* = 8.8 Hz), 3.88 (16H, t, *J* = 7.6 Hz), 1.56 (16H, quin, *J* = 7.6 Hz), 1.33 (16H, sext, *J* = 7.6 Hz), 0.90 (24H, t, *J* = 7.6 Hz); ^{13}C NMR δ_C(100 MHz; $CDCl_3$)/ppm 169.0, 143.5, 137.1, 134.5, 132.1, 128.3, 127.1, 125.1, 120.8, 94.2, 87.9, 50.0, 29.8, 20.1, 13.8; FD-LRMS *m*/*z* 1022.47 (M^{2+}, 30%), 1022.97 ($[M+1]^{2+}$, 52), 1023.47 ($[M+2]^{2+}$, 49), 1023.97 ($[M+3]^{2+}$, 25), 1024.48 ($[M+4]^{2+}$, 12), 1024.97 ($[M+5]^{2+}$, 5), 2044.95 (M^+, 60), 2045.96 ($[M+1]^+$, 100), 2046.96 ($[M+2]^+$, 84), 2047.96 ($[M+3]^+$, 46), 2048.98

([M+4]$^+$, 20), 2049.99 ([M+5]$^+$, 8); FD-HRMS Found: 2044.95260. Calc. for $C_{140}H_{124}N_8O_8$: 2044.95421.

Preparation of (*R*)$_4$-**3**, (*R*)$_4$(S_p)-**4** and (*R*)$_4$(R_p)-**5** [X = *n*Bu, Y = (*R*)-C*HMe(cHex)]

To a solution of **13b** (264 mg, 0.111 mmol) in THF (45 mL) were added 60% NaH in oil (351 mg, 8.78 mmol) and MeOH (3.1 mL) at room temperature, and the mixture was stirred for 5 min, and then diluted with dichloromethane. The organic layer was washed with aq. $NaHCO_3$, dried over magnesium sulfate, and then purified by column chromatography on SiO_2 (ethyl acetate/dichloromethane) to give **14b** (132 mg) as a yellow solid in 59% yield, which was immediately subjected to the next reaction.

To a solution of **14b** (132 mg, 0.0659 mmol) in toluene (26 mL) and THF (13 mL) containing Et_3N (0.28 mL, 2.0 mmol) was added terephthaloyl chloride in five portions (79+80+82+82+80 mg, total 2.0 mmol) at 99-101 °C over a period of 3.5 hours. After removal of a solid by filtration, the filtrate was concentrated. The residue was dissolved in chloroform along with SiO_2, which was subjected to column chromatography on SiO_2 (ethyl acetate/dichloromethane), followed by GPC (chloroform) to give a mixture of (*R*)$_4$-**3**, (*R*)$_4$(S_p)-**4** and (*R*)$_4$(R_p)-**5** (106 mg) as a pale yellow solid in 71% yield. HPLC separation (2:3 ethyl acetate/chloroform) gave (*R*)$_4$-**3** and (*R*)$_4$(S_p)-**4** in pure form, and (*R*)$_4$(R_p)-**5** as a mixture containing less than 6% (*R*)$_4$(S_p)-**4**. Each analytical sample was obtained as a pale yellow solid by reprecipitation in ethyl acetate with hexane.
(*R*)$_4$-**3**: mp >300 °C; $[\alpha]_D^{23}$ –320 (*c* 1.19 × 10^{-1} in $CHCl_3$); UV λ_{max}(CH_2Cl_2)/nm 315 (log ε 5.15); CD λ(CH_2Cl_2)/nm 387 ($\Delta\varepsilon$ –10.3), 356 (+5.3), 318 (–105) and 281 (+33.3); IR (KBr) ν_{max}/cm^{-1} 3040, 2928, 2852, 2203, 1654, 1600, 1512, 1383, 1319, 1294, 832, 730; ^{1}H NMR δ_H(400 MHz; $CDCl_3$; Me_4Si)/ppm 7.44 (2H, s), 7.41 (2H, s), 7.30 (4+4H, d, *J* = 8.4 Hz), 7.17 (4H, d, *J* = 8.4 Hz), 7.17 (4H, d, *J* = 8.8 Hz), 7.12 (8H, s), 7.09 (8H, s), 6.79 (4H, d, *J* = 8.8 Hz), 6.74 (4H, d, *J* = 8.4 Hz), 6.74 (4H, d, *J* = 8.8 Hz), 6.70 (4H, d, *J* = 8.4 Hz), 4.54 (2H, dq, *J* = 6.8, 9.6 Hz), 4.33 (2H, dq, *J* = 6.8, 9.6 Hz), 3.92-3.78 (8H, m), 2.09 (2H, br d), 2.06 (2H, br d), 1.87-1.45 (20+8H, br m), 1.39-0.86 (20+8H, m), 1.15 (6H, d, *J* = 6.8 Hz), 1.02 (6H, d, *J* = 6.8 Hz), 0.92 (6H, t, *J* = 7.2 Hz), 0.89 (6H, t, *J* = 7.2 Hz); ^{13}C NMR δ_C(100 MHz; $CDCl_3$)/ppm 170.0, 170.0, 168.9, 168.8, 143.7, 143.6, 142.4, 141.6, 138.1, 138.0, 137.1, 137.1, 136.0, 134.8, 132.1, 131.8, 131.8, 129.2, 129.1, 128.3, 127.7, 127.0, 126.7, 125.2, 125.0, 124.8, 124.6, 121.3, 121.2, 120.8, 120.7, 94.5, 94.2, 94.2, 94.0, 88.9, 88.7, 88.6, 88.5, 60.0, 58.2, 50.2, 50.2, 42.0, 41.5, 30.8, 30.6, 30.5, 30.5, 29.9, 29.7, 26.2, 26.2, 26.1, 26.0, 20.2, 20.1, 16.8, 16.6, 13.8, 13.8; FD-LRMS *m/z* 1130.54 (M^{2+}, 14%), 1131.04 ([M+1]$^{2+}$, 25), 1131.54 ([M+2]$^{2+}$, 24), 1132.04 ([M+3]$^{2+}$, 15), 1132.54 ([M+4]$^{2+}$, 8), 1133.04 ([M+5]$^{2+}$, 3), 2261.08 (M^+, 56), 2262.09 ([M+1]$^+$, 100), 2263.09 ([M+2]$^+$, 91), 2264.10 ([M+3]$^+$, 55), 2265.10 ([M+4]$^+$, 26), 2266.10 ([M+5]$^+$, 11); FD-HRMS Found: 2261.14541. Calc. for $C_{156}H_{148}N_8O_8$: 2261.14201.
(*R*)$_4$(S_p)-**4**: mp 238-245 °C (dec); $[\alpha]_D^{23}$ –51.7 (*c* 5.20 × 10^{-2} in $CHCl_3$); UV λ_{max}(CH_2Cl_2)/nm 320 (log ε 5.18); CD λ(CH_2Cl_2)/nm 351 ($\Delta\varepsilon$ +21.1) and 277 (–54.2); IR (KBr) ν_{max}/cm^{-1} 3040, 2929,

2852, 2205, 1654, 1600, 1513, 1379, 1319, 1294, 833, 727; ^{1}H NMR δ_H(400 MHz; $CDCl_3$; Me_4Si)/ppm 7.48 (4H, s), 7.30 (8H, d, *J* = 8.4 Hz), 7.22 (8H, d, *J* = 8.8 Hz), 7.11 (8H, s), 7.08 (8H, s), 6.81 (8H, d, *J* = 8.4 Hz), 6.78 (8H, d, *J* = 8.8 Hz), 4.43 (4H, dq, *J* = 6.8, 9.6 Hz), 3.87 (4H, ddd, *J* = 7.6, 13.6, 22.4 Hz), 3.84 (4H, ddd, *J* = 7.6, 13.6, 22.4 Hz), 2.07 (4H, br d), 1.84-1.50 (20+8H, m), 1.36-0.87 (20+8H, m), 1.12 (12H, d, *J* = 6.8 Hz), 0.89 (12H, t, *J* = 7.2 Hz); ^{13}C NMR δ_C(100 MHz; $CDCl_3$)/ppm 170.0, 168.9, 143.5, 142.1, 138.0, 137.1, 134.2, 132.1, 131.7, 129.1, 128.3, 127.7, 126.9, 125.3, 125.2, 121.1, 120.7, 94.3, 94.1, 88.1, 87.9, 59.2, 50.1, 41.9, 30.6, 30.6, 29.7, 26.2, 26.1, 26.0, 20.1, 16.7, 13.8; FD-LRMS *m*/*z* 1130.50 (M^{2+}, 17%), 1130.99 ($[M+1]^{2+}$, 30), 1131.50 ($[M+2]^{2+}$, 28), 1131.99 ($[M+3]^{2+}$, 18), 1132.50 ($[M+4]^{2+}$, 8), 1133.00 ($[M+5]^{2+}$, 3), 2261.02 (M^+, 58), 2262.03 ($[M+1]^+$, 100), 2263.03 ($[M+2]^+$, 91), 2264.03 ($[M+3]^+$, 56), 2265.03 ($[M+4]^+$, 27), 2266.04 ($[M+5]^+$, 10); FD-HRMS Found: 2261.14531. Calc. for $C_{156}H_{148}N_8O_8$: 2261.14201.

$(R)_4(R_p)$-**5** (a mixture containing less than 6% $(R)_4(S_p)$-**4**: mp 232-235 °C (dec); $[\alpha]_D^{23}$ –105 (*c* 8.55 × 10^{-2} in $CHCl_3$); UV λ_{max}(CH_2Cl_2)/nm 322 (log ε 5.20); CD λ(CH_2Cl_2)/nm 321 ($\Delta\varepsilon$ –20.4) and 279 (+24.4); IR (KBr) ν_{max}/cm^{-1} 3040, 2929, 2852, 2205, 1655, 1600, 1513, 1379, 1319, 1294, 833, 727; ^{1}H NMR δ_H(400 MHz; $CDCl_3$; Me_4Si)/ppm 7.49 (4H, s), 7.31 (8H, d, *J* = 8.4 Hz), 7.25 (8H, d, *J* = 8.8 Hz), 7.13 (8H, s), 7.09 (8H, s), 6.84 (8H, d, *J* = 8.8 Hz), 6.80 (8H, d, *J* = 8.8 Hz), 4.23 (4H, br dq), 3.88 (4H, ddd, *J* = 7.6, 13.6, 21.6 Hz), 3.85 (4H, ddd, *J* = 7.6, 13.6, 21.6 Hz), 2.00 (4H, br d), 1.84-1.51 (20+8H, m), 1.37-1.09 (16+8H, m), 1.23 (12H, d, *J* = 6.8 Hz), 1.02-0.89 (4H, br m), 0.89 (12H, t, *J* = 7.2 Hz); ^{13}C NMR δ_C(100 MHz; $CDCl_3$)/ppm 169.7, 168.9, 143.6, 142.6, 138.0, 137.1, 134.4, 132.1, 131.8, 128.9, 128.3, 127.9, 126.9, 125.1, 121.1, 120.8, 94.3, 94.2, 88.0, 87.9, 60.9, 50.1, 40.9, 30.9, 30.6, 29.7, 26.2, 26.1, 26.0, 20.1, 16.7, 13.8; FD-LRMS *m*/*z* 1130.56 (M^{2+}, 17%), 1131.07 ($[M+1]^{2+}$, 29), 1131.57 ($[M+2]^{2+}$, 28), 1132.07 ($[M+3]^{2+}$, 18), 1132.57 ($[M+4]^{2+}$, 9), 1133.08 ($[M+5]^{2+}$, 4), 2261.14 (M^+, 56), 2262.15 ($[M+1]^+$, 100), 2263.15 ($[M+2]^+$, 95), 2264.16 ($[M+3]^+$, 59), 2265.16 ($[M+4]^+$, 29), 2266.17 ($[M+5]^+$, 12); FD-HRMS Found: 2261.13926. Calc. for $C_{156}H_{148}N_8O_8$: 2261.14201.

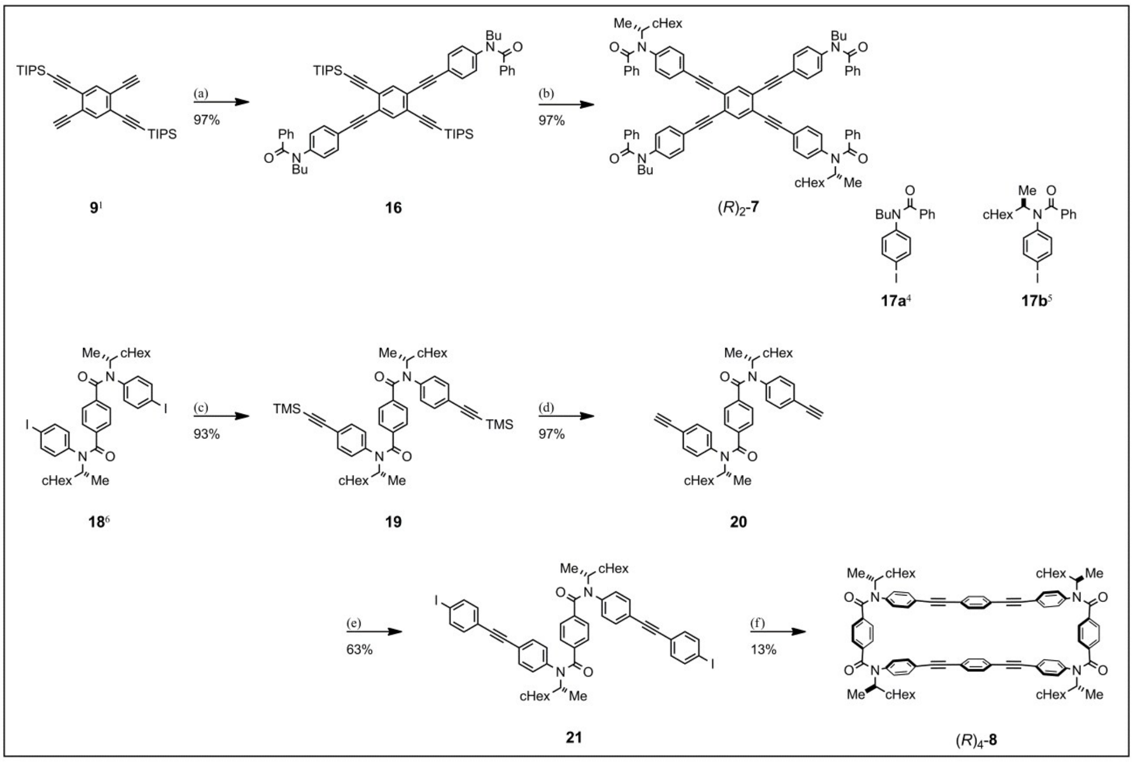

Scheme S2. Preparation of references (R)$_2$-**7** and (R)$_4$-**8**. Reagents: (a) **17a**,[4] Pd(PPh_3)$_4$, CuI, $i$$Pr_2NH$, THF; (b) **17b**,[5] Pd(PPh_3)$_4$, CuI, TBAF, Et_3N, THF; (c) TMSA, Pd(PPh_3)$_4$, CuI, Et_3N, THF; (d) K_2CO_3, MeOH, THF; (e) 1,4-diiodobenzene, Pd(PPh_3)$_4$, CuI, Et_3N, THF; (f) **20**, Pd(PPh_3)$_4$, CuI, Et_3N, toluene.

Preparation of **16**

To a solution of **9** (319 mg, 0.655 mmol) and **17a**[4] (614 mg, 1.62 mmol) in THF (5 mL) and $i$$Pr_2NH$ (5 mL) were added Pd(PPh_3)$_4$ (45 mg, 0.039 mmol) and CuI (15 mg, 0.079 mmol) at room temperature under an argon atmosphere, and the mixture was stirred at 50 ºC for 21 hours. After removal of a solid by filtration, the filtrate was concentrated and purified by column chromatography on SiO_2 (dichloromethane–ethyl acetate/dichloromethane) to give **16** (629 mg) as a pale yellow solid in 97% yield. An analytical sample was suspended in refluxed ethanol, and collected as a white solid. **16**: mp 243-244 °C; elemental analyses Found: C, 79.99; H, 8.12; N, 2.82%. Calc. for $C_{66}H_{80}N_2O_2Si_2$: C, 80.11; H, 8.15; N, 2.83%; IR (KBr) ν_{max}/cm^{-1} 3061, 2941, 2864, 2225, 2155, 1664, 1656, 1649, 1644, 1633, 1599, 1512; ^{1}H NMR δ_H(400 MHz; $CDCl_3$; Me_4Si)/ppm 7.59 (2H, s), 7.35 (4H, d, J = 8.8 Hz), 7.30-7.22 (6H, m), 7.19-7.15 (4H, m), 6.98 (4H, J = 8.8 Hz), 3.94 (4H, t, J = 7.2 Hz), 1.65-1.57 (4H, m), 1.37 (4H, sext, J = 7.2 Hz), 0.92 (6H, t, J = 7.2 Hz); ^{13}C NMR δ_C(100 MHz; $CDCl_3$)/ppm 170.2, 143.8, 136.1, 136.0, 132.5, 129.7, 128.7, 127.8, 127.4, 125.3, 125.1, 120.9, 104.1, 97.7, 94.1, 87.9, 50.1, 29.9, 20.2, 18.7, 13.8, 11.2; FD-LRMS m/z 988.59 (M$^+$, 100%), 989.59 ([M+1]$^+$, 82), 990.59 ([M+2]$^+$, 41), 991.59 ([M+3]$^+$, 16).

Preparation of $(R)_2$-**7**

To a solution of **16** (540 mg, 0.546 mmol), **17b**[5] (658 mg, 1.52 mmol), $Pd(PPh_3)_4$ (63 mg, 0.055 mmol) and CuI (20 mg, 0.10 mmol) in THF (16 mL) and Et_3N (40 mL) was added a solution of TBAF (1.2 mmol) in THF (4.6 mL) over a period of 2 hours via a syringe pump at 52 °C under an argon atmosphere, and the mixture was stirred at that temperature for 25 min. After removal of the solvents by evaporation, the residue was dissolved in ethyl acetate, which was washed with 0.1M aq. HCl, and brine, dried over magnesium sulfate, and then purified by column chromatography on SiO_2 (ethyl acetate/dichloromethane) to give $(R)_2$-**7** (681 mg) as a brownish-white solid in 97% yield. An analytical sample was suspended in refluxed ethanol, and collected as a white solid. $(R)_2$-**7**: mp 232-234 °C (dec); $[\alpha]_D^{22}$ –68.8 (c 2.47 × 10^{-1} in $CHCl_3$); elemental analyses Found: C, 83.85; H, 6.62; N, 4.34%. Calc. for $C_{90}H_{86}N_4O_4$: C, 83.95; H, 6.73; N, 4.35%; UV $\lambda_{max}(CH_2Cl_2)$/nm 375sh (log ε 4.74) and 332 (5.11); CD $\lambda(CH_2Cl_2)$/nm 390 ($\Delta\varepsilon$ +1.0), 332 (–8.3) and 280 (–1.0); IR (KBr) ν_{max}/cm^{-1} 3050, 2927, 2851, 2204, 1655, 1650, 1599, 1513; ^{1}H NMR δ_H(400 MHz; $CDCl_3$; Me_4Si)/ppm 7.65 (2H, s), 7.36-7.23 (4+10H, m), 7.35 (4H, d, J = 8.4 Hz), 7.23-7.14 (10H, m), 7.00-6.97 (4H, m), 6.99 (4H, d, J = 8.4 Hz), 4.46 (2H, br s), 3.95 (4H, t, J = 7.6 Hz), 2.16 (2H, br d), 1.89-1.66 (10H, m), 1.67-1.59 (4H, m), 1.38 (4H, sext, J = 7.6 Hz), 1.32-0.91 (10H, m), 1.23 (6H, d, J = 6.8 Hz), 0.93 (6H, t, J = 7.6 Hz); ^{13}C NMR δ_C(100 MHz; $CDCl_3$)/ppm 170.7, 170.1, 144.1, 142.7, 137.0, 136.0, 135.0, 132.4, 132.0, 129.7, 129.5, 129.3, 128.7, 128.5, 127.8, 127.8, 127.6, 125.0, 121.1, 120.7, 94.8, 94.7, 88.2, 88.0, 59.7, 50.2, 41.5, 30.8, 30.5, 29.9, 26.3, 26.1, 26.1, 20.2, 16.9, 13.8; FD-LRMS m/z 1286.62 (M^+, 100%), 1287.63 ($[M+1]^+$, 100), 1288.63 ($[M+2]^+$, 52), 1289.63 ($[M+3]^+$, 19), 1290.64 ($[M+4]^+$, 6).

Preparation of **18**[6]

A mixture of (R)-N-(1-cyclohexylethyl)-4-iodoaniline[5] (5.63 g, 17.1 mmol) and terephthaloyl chloride (1.72 g, 8.47 mmol) in toluene (120 mL) containing Et_3N (5 mL) was refluxed for 19 hours. After removal of the solvent by evaporation, the residue was purified by column chromatography on SiO_2 (dichloromethane–ethyl acetate/dichloromethane), followed by washing in refluxed methanol to give **18** (5.81 g) as a white solid in 87% yield.

Preparation of **19**

To a solution of **18** (509 mg, 0.645 mmol), $Pd(PPh_3)_4$ (22 mg, 0.019 mmol) and CuI (9 mg, 0.05 mmol) in THF (3.5 mL) and Et_3N (3.5 mL) was added trimetylsilylacetylene (0.27 mL, 1.9 mmol) at room temperature under an argon atmosphere, and the mixture was stirred at 40 ºC for 45 min. After removal of a solid by filtration, the filtrate was concentrated and purified by column chromatography on SiO_2 (dichloromethane–ethyl acetate/dichloromethane) to give **19** (436 mg) as a white amorphous solid in 93% yield. An analytical sample was obtained as colorless crystals by further purification through GPC (chloroform), followed by recrystallization from ethanol. **19**: mp

142-143 °C; $[\alpha]_D^{22}$ –149 (c 6.83 × 10^{-1} in $CHCl_3$); elemental analyses Found: C, 75.53; H, 8.35; N, 3.78%. Calc. for $C_{46}H_{60}N_2O_2Si_2$: C, 75.77; H, 8.29; N, 3.84%; IR (KBr) ν_{max}/cm^{-1} 3041, 2928, 2851, 2156, 1649, 1599, 1504, 1319, 863, 843; ^{1}H NMR δ_H(400 MHz; $CDCl_3$; Me_4Si)/ppm 7.27 (4H, br d), 6.97 (4H, br s), 6.80 (4H, br d), 4.45 (2H, br s), 2.06 (2H, br d), 1.84-1.52 (10H, br m), 1.28-1.05 (8H, br m), 1.10 (6H, d, J = 7.2 Hz), 1.05-0.89 (2H, br m), 0.24 (18H, s); ^{13}C NMR δ_C(100 MHz; $CDCl_3$)/ppm 170.0, 141.4, 137.7, 132.4, 129.3, 127.6, 121.8, 104.0, 95.7, 58.7, 41.4, 30.7, 30.3, 26.2, 26.1, 26.0, 16.7, -0.1; FD-LRMS m/z 728.45 (M^+, 100%), 729.45 ($[M+1]^+$, 70), 730.45 ($[M+2]^+$, 30), 731.45 ($[M+3]^+$, 9).

Preparation of **20**

A mixture of **19** (306 mg, 0.420 mmol), K_2CO_3 (120 mg, 0.870 mmol), MeOH (1 mL) and THF (3 mL) was stirred at room temperature for 40 min. After removal of a solid by filtration, the filtrate was concentrated and purified by column chromatography on SiO_2 (dichloromethane–ethyl acetate/dichloromethane) to give **20** (237 mg) as a white solid in 97% yield. An analytical sample was obtained as colorless crystals by further purification through GPC (chloroform), followed by recrystallization from 1:9 ethyl acetate/hexane. **20**: mp 192-193 °C; $[\alpha]_D^{22}$ –176 (c 2.88 × 10^{-1} in $CHCl_3$); elemental analyses Found: C, 82.27; H, 7.71; N, 4.78%. Calc. for $C_{40}H_{44}N_2O_2$: C, 82.15; H, 7.58; N, 4.79%; IR (KBr) ν_{max}/cm^{-1} 3277, 3038, 2966, 2924, 2851, 2108, 1641, 1601, 1504; ^{1}H NMR δ_H(400 MHz; $CDCl_3$; Me_4Si)/ppm 7.30 (4H, d, J = 8.4 Hz), 7.00 (4H, s), 6.82 (4H, br d), 4.43 (2H, br s), 3.12 (2H, s), 2.06 (2H, br d), 1.80-1.59 (8H, br m), 1.29-1.08 (10H, br m), 1.12 (6H, t, J = 7.2 Hz), 1.03-0.93 (2H, br m); ^{13}C NMR δ_C(100 MHz; $CDCl_3$)/ppm 170.0, 137.8, 132.6, 129.4, 127.7, 120.8, 82.6, 78.5, 59.0, 41.4, 30.8, 30.4, 26.2, 26.1, 26.0, 16.7; FD-LRMS m/z 584.35 (M^+, 100%), 585.36 ($[M+1]^+$, 49), 586.36 ($[M+2]^+$, 12).

Preparation of **21**

To a suspension of 1,4-diiodobenzene (939 mg, 2.85 mmol), $Pd(PPh_3)_4$ (16 mg, 0.014 mmol) and CuI (5 mg, 0.03 mmol) in Et_3N (4 mL) was added a solution of **20** (208 mg, 0.356 mmol) in THF (3.5 mL) over a period of 3 hours via a syringe pump at 41 °C under an argon atmosphere, and the mixture was stirred at that temperature for 20 min. After removal of the solvents by evaporation, the residue was purified by column chromatography on SiO_2 (dichloromethane–ethyl acetate/dichloromethane) to give **21** (222 mg) as a pale yellow solid in 63% yield, which was further purified by GPC (chloroform). An analytical sample was obtained as a white solid by reprecipitation from 1:5 chloroform/acetone. **21**: mp 284-285 °C; $[\alpha]_D^{23}$ –143 (c 4.95 × 10^{-1} in $CHCl_3$); elemental analyses Found: C, 62.90; H, 4.95; N, 2.78%. Calc. for $C_{52}H_{50}I_2N_2O_2$: C, 63.16; H, 5.10; N, 2.83%; IR (KBr) ν_{max}/cm^{-1} 3043, 2927, 2848, 2216, 1655, 1643, 1632, 1600, 1509; ^{1}H NMR δ_H(400 MHz; $CDCl_3$; Me_4Si)/ppm 7.65 (4H, d, J = 8.4 Hz), 7.34 (4H, br d), 7.22 (4H, d, J = 8.4 Hz), 7.00 (4H, br s), 6.84 (4H, br d), 4.45 (2H, br s), 2.08 (2H, br d), 1.82-1.54 (10H, br m), 1.31-1.06 (8H, br m), 1.13

(6H, d, J = 7.2 Hz), 1.06-0.89 (2H, br m); ^{13}C NMR δ_C(100 MHz; $CDCl_3$)/ppm 170.1, 141.4, 137.9, 137.6, 133.0, 132.0, 129.5, 127.6, 122.3, 121.5, 94.5, 89.9, 89.8, 58.8, 41.4, 30.8, 30.3, 26.2, 26.1, 26.0, 16.8; FD-LRMS m/z 988.22 (M^+, 100%), 989.22 ($[M+1]^+$, 58), 990.22 ($[M+2]^+$, 18).

Preparation of (R)$_4$-**8**

To a solution of **20** (69 mg, 0.12 mmol) and **21** (100 mg, 0.101 mmol) in Et_3N (20 mL) and toluene (20 mL) were added $Pd(PPh_3)_4$ (17 mg, 0.015 mmol) and CuI (5 mg, 0.03 mmol) at 70 °C under an argon atmosphere, and the mixture was stirred at 80 ºC for 1.5 hours. After removal of the solvents by evaporation, the residue was dissolved in dichloromethane, which was washed with 0.2N aq. HCl and brine, dried over magnesium sulfate, and then purified by column chromatography on SiO_2 (ethyl acetate/dichloromethane), followed by GPC (chloroform) to give (R)$_4$-**8** (17 mg) as a pale-yellowish-white solid in 13% yield. An analytical sample was suspended in chloroform and acetone, and collected as a pale-yellowish-white solid. (R)$_4$-**8**: mp >300 °C; $[\alpha]_D^{23}$ –361 (c 1.30 × 10^{-1} in $CHCl_3$); elemental analyses Found: C, 83.64; H, 7.20; N, 4.20%. Calc. for $C_{92}H_{92}N_4O_4$: C, 83.85; H, 7.04; N, 4.25%; UV λ_{max}(CH_2Cl_2)/nm 309 (log ε 4.97); CD λ(CH_2Cl_2)/nm 336 ($\Delta\varepsilon$ –28.0), 308 (–8.4), 286 (–19.5) and 267 (+1.0); IR (KBr) ν_{max}/cm^{-1} 3038, 2928, 2851, 2212, 1654, 1650, 1599, 1517; ^{1}H NMR δ_H(400 MHz; $CDCl_3$; Me_4Si)/ppm 7.31 (8H, d, J = 8.4 Hz), 7.14 (8H, s), 7.06 (8H, s), 6.82 (8H, d, J = 8.4 Hz), 4.46 (4H, dq, J = 6.8, 9.6 Hz), 2.09 (4H, br d), 1.86-1.55 (20H, m), 1.32-1.06 (16H, m), 1.12 (12H, d, J = 6.8 Hz), 1.02-0.94 (4H, m); ^{13}C NMR δ_C(100 MHz; $CDCl_3$)/ppm 170.3, 141.5, 138.2, 131.8, 131.2, 129.4, 127.4, 122.7, 121.4, 90.1, 89.9, 58.8, 41.5, 30.7, 30.4, 26.2, 26.1, 26.0, 16.7; FD-LRMS m/z 1316.67 (M^+, 95%), 1317.68 ($[M+1]^+$, 100), 1318.68 ($[M+2]^+$, 51), 1319.68 ($[M+3]^+$, 20), 1320.70 ($[M+4]^+$, 7).

References

1 J. A. Marsden, J. J. Miller, L. D. Shirtcliff and M. M. Haley, *J. Am. Chem. Soc.*, 2005, **127**, 2464.

2 R. Katoono, K. Kusaka, K. Fujiwara and T. Suzuki, *Chem. Asian J.*, 2014, **9**, 3182.

3 R. Katoono and T. Suzuki, *Chem. Commun.*, 2016, DOI: 10.1039/C5CC09069E.

4 R. Katoono, K. Fujiwara and T. Suzuki, *Chem. Commun.*, 2014, **50**, 5438.

5 R. Katoono, H. Kawai, M. Ohkita, K. Fujiwara and T. Suzuki, *Chem. Commun.*, 2013, **49**, 10352.

6 R. Katoono, Y. Tanaka, K. Fujiwara and T. Suzuki, *J. Org. Chem.*, 2014, **79**, 10218.
